# Genomic and phenotypic characterization of myxoma virus from Great Britain reveals multiple evolutionary pathways distinct from those in Australia

**DOI:** 10.1371/journal.ppat.1006252

**Published:** 2017-03-02

**Authors:** Peter J. Kerr, Isabella M. Cattadori, Matthew B. Rogers, Adam Fitch, Adam Geber, June Liu, Derek G. Sim, Brian Boag, John-Sebastian Eden, Elodie Ghedin, Andrew F. Read, Edward C. Holmes

**Affiliations:** 1 Marie Bashir Institute for Infectious Diseases and Biosecurity, School of Life and Environmental Sciences and Sydney Medical School, University of Sydney, Sydney, New South Wales 2006, Australia; 2 CSIRO Health and Biosecurity, Canberra, Australian Capital Territory 2601, Australia; 3 Center for Infectious Disease Dynamics and Department of Biology, The Pennsylvania State University, University Park, Pennsylvania 16802, United States of America; 4 University of Pittsburgh School of Medicine, Pittsburgh, Pennsylvania 15261, United States of America; 5 Center for Genomics & Systems Biology, Department of Biology, New York University, New York, New York 10003, United States of America; 6 The James Hutton Institute, Invergowrie, DD2 5DA, United Kingdom; 7 Department of Entomology, The Pennsylvania State University, University Park, Pennsylvania 16802, United States of America; University of Glasgow, UNITED KINGDOM

## Abstract

The co-evolution of myxoma virus (MYXV) and the European rabbit occurred independently in Australia and Europe from different progenitor viruses. Although this is the canonical study of the evolution of virulence, whether the genomic and phenotypic outcomes of MYXV evolution in Europe mirror those observed in Australia is unknown. We addressed this question using viruses isolated in the United Kingdom early in the MYXV epizootic (1954–1955) and between 2008–2013. The later UK viruses fell into three distinct lineages indicative of a long period of separation and independent evolution. Although rates of evolutionary change were almost identical to those previously described for MYXV in Australia and strongly clock-like, genome evolution in the UK and Australia showed little convergence. The phenotypes of eight UK viruses from three lineages were characterized in laboratory rabbits and compared to the progenitor (release) Lausanne strain. Inferred virulence ranged from highly virulent (grade 1) to highly attenuated (grade 5). Two broad disease types were seen: cutaneous nodular myxomatosis characterized by multiple raised secondary cutaneous lesions, or an amyxomatous phenotype with few or no secondary lesions. A novel clinical outcome was acute death with pulmonary oedema and haemorrhage, often associated with bacteria in many tissues but an absence of inflammatory cells. Notably, reading frame disruptions in genes defined as essential for virulence in the progenitor Lausanne strain were compatible with the acquisition of high virulence. Combined, these data support a model of ongoing host-pathogen co-evolution in which multiple genetic pathways can produce successful outcomes in the field that involve both different virulence grades and disease phenotypes, with alterations in tissue tropism and disease mechanisms.

## Author summary

Species jumps and subsequent pathogen evolution are of increasing importance in a globally connected world. The co-evolution of myxoma virus and the European rabbit following the introduction of the virus into Australia in 1950 is the canonical case of host jumping and host-pathogen co-evolution on a continental scale. This natural experiment was repeated with the release of a separate strain of myxoma virus in Europe. On both continents moderately attenuated strains of virus became dominant while rabbits were selected for resistance to myxomatosis. Here we examine the genotypic and phenotypic evolution of myxoma virus in Great Britain compared to Australia and show that despite ecological convergence and equivalent evolutionary rates, the virus has followed distinct evolutionary pathways on both continents with few shared mutations. Furthermore, we reveal novel mechanisms of pathogenesis and tissue tropism compared to the progenitor virus, and that the disruption of virulence genes is compatible with high virulence. This suggests that mutations have occurred that can compensate for the loss of virulence genes driven by the nexus between virulence and transmission in an ongoing host-pathogen arms race.

## Introduction

The establishment and spread of *Myxoma viru*s (MYXV; genus *Leporipoxvirus;* family *Poxviridae*) in the wild European rabbit (*Oryctolagus cuniculus*) population of Australia in 1950 initiated the textbook case study of host-pathogen co-evolution on a continental scale [1, 2]. The virus was novel to the European rabbit having evolved in the Brazilian tapeti (*Sylvilagus brasiliensis*). In the tapeti MYXV induces an innocuous, localized cutaneous fibroma from which the virus is mechanically transmitted by mosquitoes or fleas. However, MYXV proteins that had evolved to suppress immune clearance and facilitate virus persistence in the natural host overwhelmed the immune system of the European rabbit causing the disseminated, lethal disease myxomatosis [2, 3].

In Australia MYXV was released into naïve rabbit populations as a biocontrol agent. The initial virus, a strain known as SLS with a case fatality rate (CFR) estimated at 99.8% [4], was rapidly replaced by moderately attenuated viruses, which by permitting longer survival of the infected rabbit were more likely to be transmitted by mosquitoes. The majority of these attenuated viruses still maintained relatively high CFRs of 70–95% [5, 6]. Simultaneously, there was very strong selection pressure for the evolution of genetically resistant rabbits [7, 8]. It is likely that the increased resistance in the rabbit population also drove selection for increased virulence in the virus to maintain transmissibility, as highly attenuated viruses transmitted poorly [9, 10, 11].

This large-scale evolutionary “experiment” is especially informative because it was repeated on a continental scale as MYXV was subsequently released in Europe. In June 1952, a landholder in France inoculated two wild rabbits with a strain of MYXV (Brazil Campinas/1949), now termed the Lausanne (Lu) strain. From this starting point, MYXV spread through the wild and domestic rabbit populations of Europe [12]. Myxomatosis was detected in wild rabbits in Britain in October 1953, probably due to the illegal release of an infected rabbit from France [13]. Despite attempts at control, the virus became established and spread throughout the wild rabbit population [14], which was eventually reduced to perhaps 1% of the pre-myxomatosis level. Strikingly, although the European release involved a different starting strain, with different insect vectors and ecological conditions, it resulted in essentially the same outcome in terms of virulence evolution [1, 12].

To facilitate evolutionary studies, field isolates of MYXV were classified into virulence grades from 1 to 5 based on average survival times (AST) in small groups of laboratory rabbits. The progenitor type viruses, killing 100% of infected rabbits, were of grade 1 virulence, while grade 5 viruses were highly attenuated with CFRs <50%. Most field isolates collected following the initial radiation in Australia were of grade 3 virulence with CFRs of 70–95% [5, 6]. The grade 3 classification was later split into grade 3A and 3B to provide greater resolution [15]. Although the initial virus isolates in Britain were of grade 1 virulence [5], attenuated viruses were detected within 12 months [16, 5].

A large scale study of the virulence of UK MYXV isolates from 1962 revealed a similar evolutionary pattern to Australia, with the majority of isolates being of grade 3 virulence [15]. Studies of UK MYXV isolates from 1975 and 1981 confirmed the predominance of grade 3 viruses, but also showed that grade 2 viruses (with CFRs of >95%) had become much more common than in Australia; over 90% of viruses tested in 1981 were grade 3A or grade 2, implying CFRs of >90% [17]. Genetic resistance to MYXV was documented much later in Britain than in Australia, but then rapidly increased in the wild rabbit population [18, 19] and may again have driven selection for higher virulence.

Although there have been detailed studies of the ecology, transmission, virulence and resistance of MYXV in Britain, little is known about the genetic and phenotypic basis of MYXV evolution and whether and how it parallels the evolutionary process seen in Australia. Indeed, previous studies have largely focused on early virus isolates sampled between 1954 and 1955 [20, 21]. To address this central question in viral evolution we determined the genome sequences of 21 MYXV isolates sampled between 2008 and 2014 in Scotland and England. Importantly, we characterise the phenotype of a number of these viruses in laboratory rabbits compared to the progenitor Lu strain and reveal major changes in disease pathogenesis.

## Results

### Diversity and evolution of MYXV in Europe

The prototype Lu sequence [22, 23] consists of 161,777 nucleotides of double-stranded DNA with closed single stranded hairpin loops at the termini and duplicated terminal inverted repeats (TIRs) of 11,577 bp. The virus encodes 158 unique open reading frames (ORFs), 12 of which are duplicated in the TIRs.

**Fig 1:**
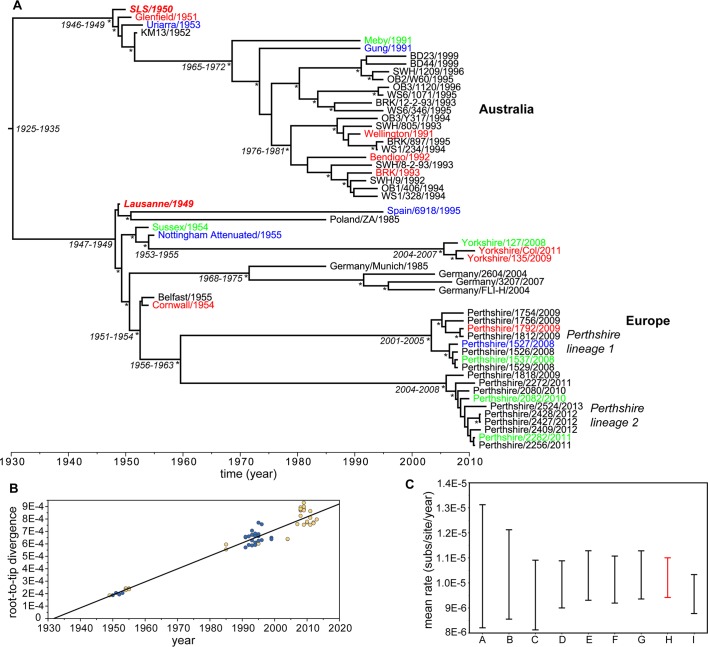
Evolutionary history of MXYV. (A) Maximum clade credibility (MCC) tree of 57 isolates of MYXV from the Australian and European epizootics including a sequence from Spain [23], four from Germany and one from Poland [24]. Sequence labels are color-coded to reflect virulence grade: grade 1, 2 = red, grade 3 = green, grade 4–5 = blue, non-quantified grade = black. The Lausanne and SLS progenitor strains are shown in bold italic. Tip times reflect the year of sampling. Estimated times to common ancestry are shown for key nodes and posterior probability values greater than 0.95 are marked by the * symbol. The different lineages of UK lineages are marked. (B) Regression of root-to-tip MYXV genetic distances against the year of sampling. Australian viruses are shaded blue and those from Europe in yellow. (C) Bayesian estimates of substitution rate utilizing different evolutionary models: A = Australian viruses, HKY+Γ nucleotide substitution model, relaxed clock, constant population size; B = Australian viruses, HKY+Γ, strict clock, constant population size; C = European viruses, HKY+Γ, relaxed clock, constant population size; D = European viruses, HKY+Γ, strict clock, constant population size; E = All viruses, GTR+Γ, relaxed clock, Bayesian skyride; F = All viruses, HKY+Γ, relaxed clock, constant population size; G = All viruses, HKY+Γ, relaxed clock, Bayesian skyride; H = All viruses, HKY+Γ, strict clock, constant population size (shown in red as this was used to infer the MCC tree); I = All viruses, HKY+Γ, strict clock, Bayesian skyride.

The UK viruses descend from the Lu strain that was released into Europe as a biological control (Fig 1). The earliest sequences are from the grade 1 virulence Cornwall strain (England/Cornwall/4-54/1) isolated in April 1954 and the grade 3 Sussex strain (England/Sussex/9-54/1) from September 1954 and which quickly diverged from the introduced virus [20, 21]. This divergence is captured in a phylogenetic analysis of these viruses along with an additional early isolate (Belfast/1955) sequenced here, 21 viruses from 2008–2013 (Table 1), and a number of other European viruses (Fig 1). Notably, the viruses from Perthshire, Scotland can be divided into two lineages, with those sampled in 2008 (lineage 1) phylogenetically distinct from those present in 2010–2013 (lineage 2). In 2009, both lineages were present in the Perthshire population and it is possible that our limited sampling has not detected other examples of co-circulation. Within lineage 1, the viruses sampled in 2008 are also distinct from those sampled in 2009, while there is no obvious distinction within the sequences of lineage 2 from 2009–2013. The three viruses sequenced from Yorkshire, sampled between 31/12/2008 and 8/3/2011, represent a third distinct UK lineage.

**Table 1:** Viruses sequenced in this study.

Virus designation	Sampling date
Belfast/1955	1955
Perthshire 1526	17/09/2008
Perthshire 1527	17/09/2008
Perthshire 1529	17/09/2008
Perthshire 1537	17/09/2008
Perthshire 1754	14/07/2009
Perthshire 1756	14/07/2009
Perthshire 1792	02/08/2009
Perthshire 1812	10/08/2009
Perthshire 1818	10/08/2009
Perthshire 2080	15/10/2010
Perthshire 2082	15/10/2010
Perthshire 2256	20/09/2011
Perthshire 2272	26/09/2011
Perthshire 2282	15/10/2011
Perthshire 2409	30/8/2012
Perthshire 2427	19/9/2012
Perthshire 2428	19/9/2012
Perthshire 2524	29/9/2013
Yorkshire 127	31/12/2008
Yorkshire Col	08/03/2011
Yorkshire 135	02/11/2009

Despite the difference in progenitor viruses in Australia and Europe the subsequent evolution of these viruses is strongly clock-like. Using a Bayesian approach and a strict molecular clock the mean evolutionary rate for the 32 European viruses was estimated to be 0.99 x 10^−5^ nucleotide substitutions per site, per year (subs/site/year) (95% HPD values of 0.90–1.09 x 10^−5^ subs/site/year), while the equivalent value for the 25 Australian viruses was 1.03 x 10^−5^ subs/site/year (95% HPD values = 0.86–1.21 x 10^−5^ subs/site/year). Very similar rates were obtained using a variety of data sets and nucleotide substitution, molecular clock and demographic models (Fig 1). In addition, a regression of root-to-tip genetic distance against year of sampling for the combined Australian and European data set revealed strong temporal structure (R^2^ = 0.93), with a mean evolutionary rate of 1.04 x 10^−5^ subs/site/year that was very close to that estimated using the Bayesian approach for the entire data set at 1.02 x 10^−5^ subs/site/year (95% HPD values = 0.94–1.10 x 10^−5^ subs/site/year) (Fig 1). The similarly of rates among viruses sampled on different continents suggests that their high evolutionary rate is largely a reflection of rapid background mutation as suggested for other pox viruses [25]. Under these evolutionary rates it is estimated that the two MYXV lineages from Perthshire shared a common ancestor between 1956 and 1963, while the lineage leading to the Yorkshire viruses originated between 1953 and 1955 (Fig 1).

**Fig 2:**
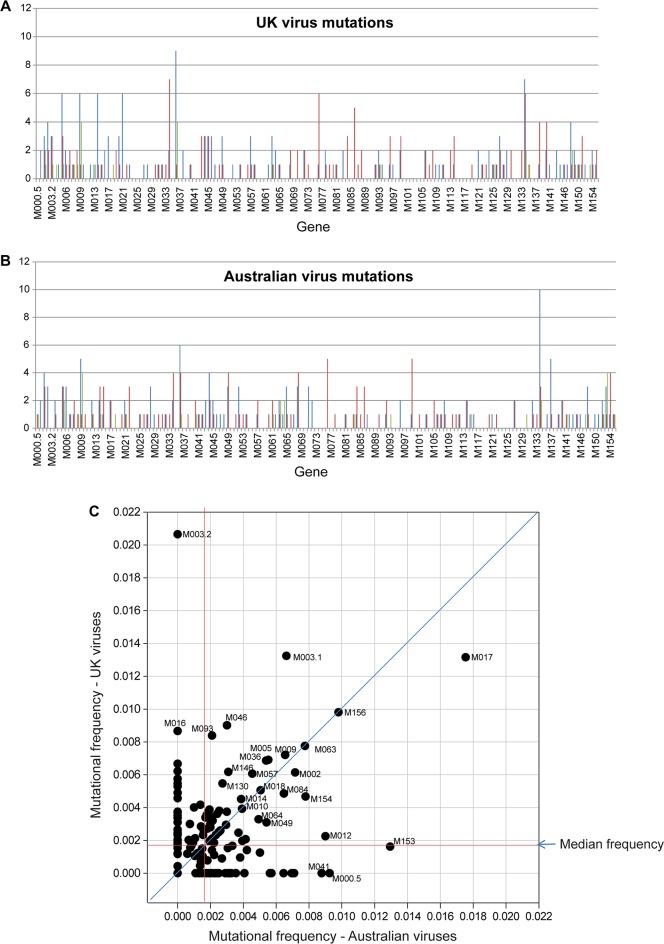
Mutation analysis in MYXV. (A) The number of mutations per gene (y-axis) in all viruses from the UK. (B) The number of mutations per gene (y-axis) in all viruses from Australia [21]. In each graph, blue lines represent the number of non-synonymous mutations, red lines represent synonymous mutations, and green lines represent indels (any insertion/deletion event was counted as a single event). (C) The total number of synonymous, non-synonymous and indel mutations per gene was standardised by dividing by the gene length. The resulting mutational frequency for each gene was plotted for UK and Australia. The red lines indicate the median mutation frequencies for the UK and Australia (which were not significantly different). The blue diagonal line indicates equal mutation frequencies for the UK and Australia. Selected individual genes are indicated.

Across all the UK viruses there were 162 non-synonymous mutations, 137 synonymous mutations and 26 insertion/deletion events within ORFs compared to Lu; 51 genes had no mutations and a further 23 only possessed synonymous changes (Fig 2A). A comparison with the mutations observed in the Australian isolates (Fig 2B) revealed that different genes tended to show the highest numbers of mutation in each case. Indeed, only the *M017L* gene exhibited frequent mutation in both data sets (Fig 2C). Overall, 23 genes contained no mutations among both the UK and Australian sequences and a further 23 had only synonymous changes (S1 Table).

As previously reported for MYXV in Australia [20, 21], single or multiple nucleotide insertions/deletions (indels) leading to the predicted disruption of ORFs were relatively common (Table 2). Disruptions of genes previously identified as having major virulence functions and leading to likely loss of function of the encoded protein occurred in *M002L/R* [26]; *M004L/R* [27, 28]; *M005L/R* [29, 30]; *M148R* [31] and *M153R* [32, 33]. In addition, there was loss of the *M009L* ORF in Perthshire lineage 1 and by two independent mutations in the Yorkshire lineage, and of the *M036L* ORF in Perthshire lineages 1 and 2. There was also an adjacent mutation in *M036L* in the early Sussex and Nottingham strains, with a possible reversal of this disruptive mutation in the Yorkshire lineage (S1 Fig). Single viruses with gene disruptions were found in all three lineages: *M135R* (Perthshire 1527) and *M008*.*1L/R* (Perthshire 2409) have been shown to have virulence functions [34, 35]. *M009L* has also been lost in most modern Australian viruses, as well as in some European isolates and in the Californian MSW strain of MYXV [20, 21, 36, 37, 24], suggesting that this gene is not essential.

**Table 2:** Gene disruptions in the UK isolates of MYXV.

Gene	Protein function	Mutation	virus
*M002L/R*	^1^Antiapoptosis/TNF inhibition	Premature stop amino acid 174; retains antiapoptosis; loses secreted TNF binding	Perthshire 1526; 1527; 1529; 1537
*M003*.*2L/R*	unknown	Premature stop amino acid 66	Perthshire 2272
*M004L/R*	Antiapoptosis	Premature stop amino acid 141; loss of C-terminal RDEL motif	Perthshire lineage 1
*M005L/R*	Antiapoptosis/E3 Ub ligase complex	Altered amino acid sequence >401 & premature stop at 471; loss of functional C-terminal F-box motif	Yorkshire lineage
*M008L/R*	E3 Ub ligase complex	Altered amino acid sequence >361 and premature stop at 421	Perthshire 2082
*M008*.*1L/R*	Secreted serine proteinase inhibitor (SERPIN)	Premature stop amino acid 268 deletes active site	Perthshire 2409
*M009L*	E3 Ub ligase complex	Premature stop amino acid 192 and amino acid 508	Perthshire lineage 1
*M009L*	E3 Ub ligase complex	Premature stop amino acid 288	Yorkshire Col; Yorkshire 135
*M009L*	E3 Ub ligase complex	Premature stop amino acid 348	Yorkshire 127
*M018L*	VACV F8L (cytoplasmic protein)	Premature stop amino acid 46 (normal 66)	Yorkshire 127
*M036L*	VACV O1L orthologue	Premature stop aa 442	Sussex (1954); Nottingham (1955)
*M036L*	VACV O1L orthologue	Premature stop amino acid 441	Perthshire lineage 1 & 2
*M036L*	VACV O1L orthologue	Premature stop amino acid 338	Perthshire lineage 2
*M134R*	Structural?	Premature stop amino acid 1953	Nottingham (1955)
*M135R*	Immunomodulatory	Premature stop amino acid 69	Perthshire 1527
*M148R*	E3 Ub ligase complex	Premature stop amino acid 170	Perthshire lineage 2
*M150R*	NF-κB inhibition; E3 Ub ligase complex	Premature stop amino acid 196	Nottingham (1955)
*M153R*	E3 Ub ligase complex/MHC-1 downregulation	Premature stop amino acid 118; loss of conserved domain	Perthshire lineage 1; Yorkshire lineage

^1^Shaded cells represent genes implicated in MYXV virulence.

In addition to indels that disrupted ORFs, there were a number of large and small indels within genes that were not disruptive (S2 Table). Moreover, there were single nucleotide indels in multiple intergenic homopolymer regions and larger deletions in some blocks of intergenic repeat sequence elements. These will not be considered further.

### Mutations in promoter regions

Temporal regulation of most MYXV genes has been predicted on the basis of conserved early, late or intermediate promoter motifs [22, 38]. However, the transcription start sites of most MYXV mRNAs have not been mapped and hence actual expression may differ from that assigned [39, 31, 40]. In the UK sequences, mutations upstream of the *M000*.*5L/R*, *M001L/R*, *M008*.*1L/R*, *M019L*, *M033L*, and *M153R* genes were located close to potential promoter sequences and could conceivably alter transcription [41, 42]. However, any effect was likely to be limited, with the possible exception of a mutation in the *M153R* putative promoter sequence in the Perthshire lineage 2 viruses which could conceivably decrease promoter activity. This mutation was also present in the Australian WS6 1071 virus.

### Phenotypes of virus isolates

To evaluate how the genetic divergence from the Lu progenitor has affected disease phenotypes in the UK viruses, groups of six laboratory rabbits were infected with representative viruses from Perthshire lineages 1 and 2, and all three Yorkshire lineage viruses, and their virulence and disease phenotypes compared to rabbits infected with the Lu progenitor virus.

**Fig 3:**
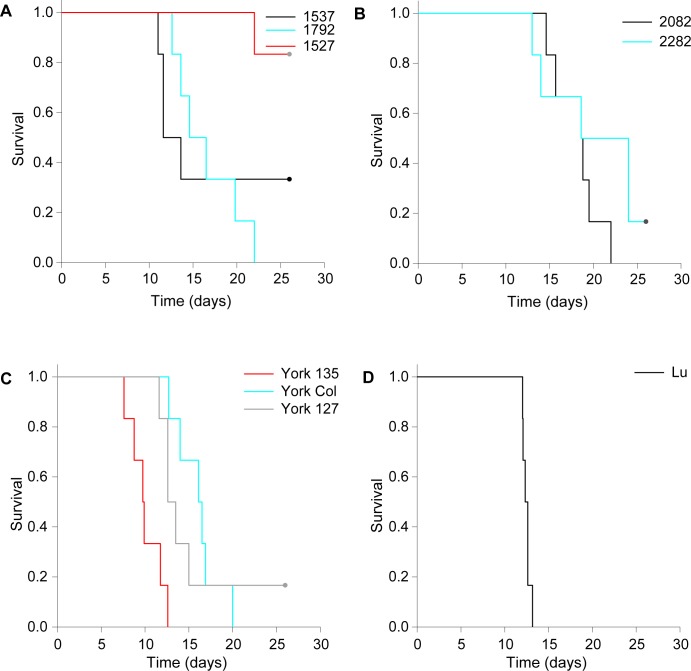
Kaplan-Meier survival plots. (A) Perthshire lineage 1. There is a statistically significant difference between Perthshire 1792 and Perthshire 1527 (p = 0.0035; log rank test), but not between Perthshire 1527 and Perthshire 1537 (p = 0.11) nor between Perthshire 1792 and Perthshire 1537 (p = 0.79). (B) Perthshire lineage 2. There is no significant difference between the two viruses studied (p = 0.25). (C) Yorkshire lineage. There is a significant difference between Yorkshire 135 and Yorkshire Col (p = 0.0015) and Yorkshire 135 and Yorkshire 127 (p = 0.019), but not between Yorkshire col and Yorkshire 127 (p = 0.53). (D) Lausanne. There is a significant difference in survival time between Yorkshire 135 and Lu (p = 0.013).

The virulence grade of each isolate was estimated using the method of Fenner and Marshall (1957) [5]. These virulence assignments were necessarily inferred since rabbits were euthanized and survival times (ST) estimated rather than using death as an endpoint (Table 3). Kaplan-Meier plots show the actual ST estimates rather than the normalized values (Fig 3). The Lu strain was tested as a control and had a similar AST to previous reports [5]. Notably, the grade 1 Yorkshire 135 isolate had a significantly lower ST than all other viruses tested including Lu.

**Table 3:** Clinical phenotypes of UK MYXV isolates.

Virus	Normalized average survival time (AST) days; (case fatality rate; CFR)	Unnormalized survival time estimates (range) days	Inferred virulence grade^1^	Disease phenotype
Lausanne	12.5 (6/6)	12.1–13.2	1	nodular
Perthshire 1792	15.9 (6/6)	12.6–22	2	amyxomatous
Yorkshire Col	15.7 (6/6)	12.7–20	2	amyxomatous
Perthshire 2082	17.9 (6/6)	14.6–22 days	3A	amyxomatous
Perthshire 2282	20.7 (5/6)	13–>26 (S?)^2^	3A	amyxomatous
Yorkshire 127	15.3s (5/6)	11.6 –S^3^	2/3A^4^	nodular
Yorkshire 135	9.1 (6/6)	7.6–12.6	1	amyxomatous
Perthshire 1537	17.1 (4/6)	11–>26 (S? S?)	3A/4^4^	amyxomatous or nodular
Perthshire 1527	n/a (1/6)	n/a	5	nodular

^1^ [43].

^2^ possible survivor.

^3^ recovered.

^4^ based on AST/CFR.

In our animal experiments the disease caused by Lu was indistinguishable from previous descriptions of Lu as the prototype European virus [5], with the exception that we did not see the copious nasal discharge, likely because of the absence of *Pasteurella multocida* in the upper respiratory tract of the specific-pathogen-free rabbits. Notable features of Lu compared to the infections with the recent virus isolates were extreme swelling of the eyelids and lips, large size of the primary lesion, large numbers of secondary cutaneous lesions and a precipitous clinical decline between days 10 and 12 (S3 Table; S4 Table).

**Fig 4:**
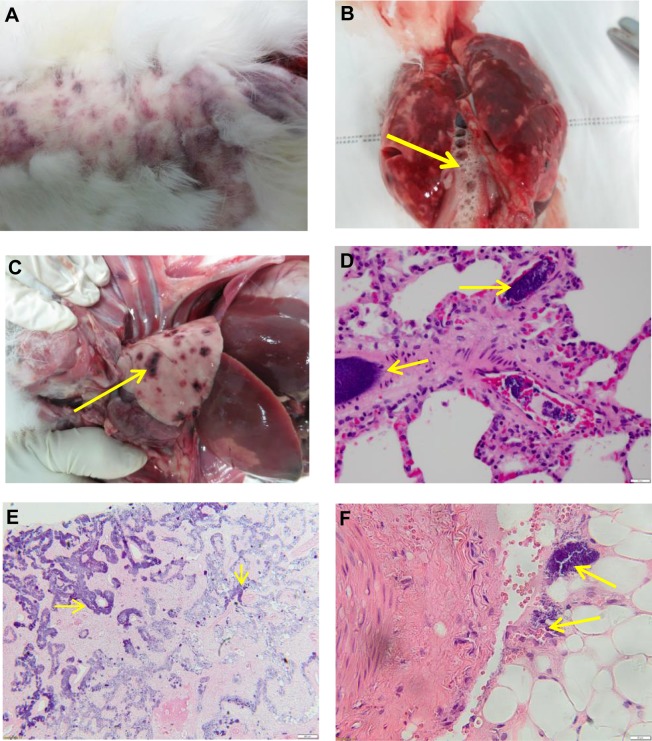
Acute collapse syndrome. (A) Epidermal hemorrhages 1–2 cm in diameter developed over 4–5 hours in the epidermis (Yorkshire Col day 12). (B) Severe pulmonary oedema with fluid and froth filling the trachea (arrowed) and bronchi and swollen wet lungs (Perthshire 2082 day 15). (C) Hemorrhages (arrowed) in lungs (Perthshire 2082 day 14). (D) Bacteria (arrows) in pulmonary blood vessels (Perthshire 2082 day 15; scale bar 20 μm). (E) Popliteal lymph node showing complete loss of lymphocytes and massive numbers of bacteria (arrows) staining purple throughout the sinuses (Perthshire 2282 day 12; scale bar 200 μm). (F) Hind leg muscle showing bacteria (arrows) in blood vessels (Perthshire 2282 day 12; scale bar 20 μm).

A striking feature of infection with some viruses from all three recent UK lineages was acute collapse resembling septic shock with relatively mild signs of myxomatosis. This was distinct from the disease caused by Lu. Hemorrhages in multiple tissues, massive pulmonary oedema and swollen, pale or granular livers were also frequently but not universally present, although the degree of pathology may have depended on timing of euthanasia or death. Aggregates of coccoid bacteria were often present in multiple tissues but with no apparent cellular inflammatory response (Fig 4; S5 Table). These rabbits often had higher virus titres in liver and lung compared to rabbits infected with Lu (S6 Table).

**Fig 5:**
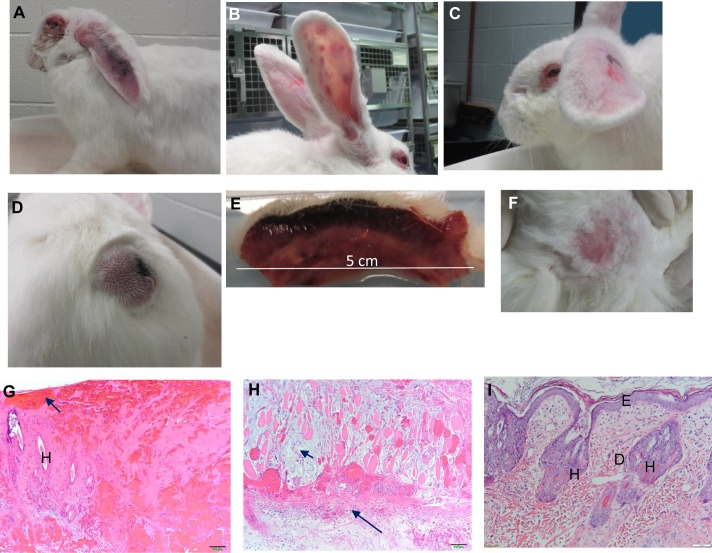
Nodular and amyxomatous phenotypes. (A) Lu day 10: grossly swollen almost granulomatous eyelids and swollen drooping ears; note the large swelling at the base of the ears. (B) Perthshire 1527 day 10; secondary lesions on ears but otherwise mild clinical signs with this grade 5 virus. (C) Perthshire 1537 day 10: moderately swollen ears, eyelids and head. Despite the alert appearance and mild clinical signs, the rabbit died with acute collapse less than 24 hours later. (D) Lu day 10: domed primary lesion oozing at top. (E) Lu day 12: section through primary lesion. (F) Perthshire 1792 day 10: amyxomatous phenotype showing very limited reaction at inoculation site. (G) Lu: histology of upper part of primary lesion day 12. Destruction of epidermis and dermis with scab formation and hemorrhage (arrowed); H: remnant hair follicle. (H) Lu primary lesion–deeper within the same lesion; short arrow indicates blue-grey staining mucinous material; long arrow indicates muscle necrosis and inflammatory cells (neutrophils). (I) Perthshire 2282 day 14: histopathology of primary lesion; note relatively normal architecture with some hyperplasia of epidermis and disruption of collagen fibres in dermis. E: epidermis; D: dermis; H: hair follicle. Scale bars = 100 μm.

Overall, disease phenotypes could be divided into: (i) a nodular cutaneous or “myxomatous” disease with prominent primary lesions at the inoculation site and secondary cutaneous lesions on ears, head, body and legs as seen with Lu, Perthshire 1527 and Yorkshire 127 viruses, or (ii) a disease that resembled the “amyxomatous” phenotype described in Europe [44, 45, 46] and characterized by a poorly defined primary lesion and no or very few secondary cutaneous lesions. This second phenotype was seen with Perthshire 1792, 2082, 2282, Yorkshire col and Yorkshire 135, while Perthshire 1537 had an intermediate phenotype (Fig 5; S4 Table; S5 Table). Acute collapse was only seen with the amyxomatous infections. Other features of myxomatosis such as swollen heads, ears, eyelids and perineum were, to some degree, common to all infections. Prolonged incubation periods described for some amyxomatous viruses [44] were not seen.

Distinctive differences were also present in the pathology of the acute collapse amyxomatous infections compared with Lu and the myxomatous phenotype (S5 Table). Bacteraemia was not a feature of the Lu infections. Although bacteria were observed in a necrotic focus in the liver of one rabbit infected with Lu, these were associated with an acute inflammatory response. The large numbers of neutrophils seen deep in cutaneous tissues and within lymph nodes in the Lu infections (Fig 5H) were absent in rabbits with the acute collapse syndrome and lymph nodes and spleens tended to be more depleted of lymphocytes in these rabbits. Late clinical signs in longer surviving or recovering rabbits were fairly typical of those described for myxomatosis caused by moderately attenuated viruses [5], with the exception that the amyxomatous viruses did not induce secondary lesions (S4 Table; S5 Table).

### Virus levels in the primary lesion

The prolonged duration of high virus titres in the epidermis of primary or secondary lesions or in sites such as eyelids or ears is critical for transmission by arthropod vectors [9]. In general, longitudinal biopsy samples showed that levels of virus in the primary lesions, measured by qPCR, increased over the first 10 days to > 10^8^ copies/mg and were then reasonably stable, albeit with reduced numbers of rabbits available for biopsy at later time points (S2 Fig). However, two virus infections had consistently lower virus loads: the grade 5 Perthshire 1527 and the grade 2/3 Yorkshire 127 strain. Both viruses had the nodular myxomatous phenotype and the lower loads were probably due to cell destruction in the epidermis. Despite the limited nature of the primary lesion in the amyxomatous phenotypes (Fig 5I) they had very high levels of virus. Similar results were obtained with titres measured by plaque assay on autopsy samples (S6 Table). Titres in the Lu infected rabbits were also relatively low, likely because of the highly scabbed and degenerate nature of the lesion (S6 Table; Fig 5). Biopsies were not collected from rabbits infected with Yorkshire 135 or Lu. Taken together with the histological and gross appearance of the primary lesions, these results indicate that the tissue response to the amyxomatous viruses is entirely different to that induced by Lu, but that this is not due to reduced virus replication.

### Genomic differences between viruses associated with clinical phenotype

Despite the observed differences in disease phenotype and virulence, viruses within each lineage exhibit limited sequence divergence. For example, Yorkshire 127 caused the nodular cutaneous phenotype while the closely related Yorkshire 135 and Yorkshire col caused the amyxomatous phenotype (Fig 3). All three Yorkshire viruses have lost the functional domain of the *M005L/R* gene and have disrupted *M009L* and *M153R* genes (Table 2). There are six amino acid differences between Yorkshire 135 and Yorkshire Col and seven between Yorkshire 135 and Yorkshire 127 (S7 Table).

The Perthshire lineage 1 viruses are more complicated, as the 2008 viruses (1527, grade 5 and 1537, grade 3/4) have a disrupted *M002L/R* gene and Perthshire 1527 has a disrupted *M135R* gene; both are virulence determinants in Lu [34]. These genes are intact in the amyxomatous 2009 Perthshire 1792 virus (grade 2). As with the Yorkshire lineage, these viruses only differ at a small number of amino acid sites (S8 Table).

Both Perthshire lineage 2 viruses tested had the amyxomatous phenotype and were of grade 3 virulence. Apart from the premature termination of M008L/R in 2082, there are only four amino acid differences between these viruses (S9 Table). Phenotypically, it was difficult to differentiate these grade 3 viruses from the grade 2 Perthshire 1792 and Yorkshire Col.

Overall, these results suggest that single amino acid changes can have a major impact on disease phenotype and virulence gene disruption may be compensated by epistatic mutations or other mechanisms.

## Discussion

Our genome-scale evolutionary analysis reveals that multiple lineages of MYXV have circulated in UK rabbits. In particular, the single lineage of viruses from Yorkshire and the two lineages present in Perthshire clearly diverged relatively early in the epizootic and have evolved independently ever since. This separation of the English and Scottish viruses could reflect a simple biogeographic division and a lack of virus gene flow, particularly since the European rabbit flea (*Spilopsyllus cuniculi*) is the main arthropod vector in the UK so that virus spread depends on movement of rabbits carrying fleas [47, 48]. However, the phylogenetic separation between the two Scottish lineages is harder to explain as they were sampled within three kilometres of each other. Because these two lineages differ in the range of temporal sampling (2008–2009) and (2009–2013) it is possible that the later sampled lineage is a more recent invader into the study area and has outcompeted the previously existing lineage. Anecdotally, in 2009 this study site experienced a high mortality of rabbits due to myxomatosis, compatible with the possible invasion of a new strain into the area.

Importantly, our comparison of MYXV genome sequences from the UK and Australia confirms previous conclusions that there is no single pathway to attenuation from the progenitor viruses or from attenuation back to virulence [20]. Indeed, it is striking that there are almost no shared mutations between the viruses from the two radiations despite the large number of complete genomes now sequenced. Hence, evolutionary success in these large genome DNA viruses has clearly resulted from the exploration of multiple evolutionary pathways along which different disease phenotypes appear. Indeed, our animal trials reveal that the clinical phenotype of a number of the UK viruses showed dramatic changes compared to the progenitor Lu virus, as well as within and between the modern viral lineages.

Generalized disease seems critical for efficient virus transmission in European rabbits, with rabbits that survive infection (and therefore control virus replication) being poor transmitters [10]. In addition, resistance is manifest as control of virus replication rather than prevention of infection [49, 50, 51], so is likely to select for virus mutations that can overcome this control. The emergence of genetic resistance in the wild rabbit population likely shifted selection towards more virulent viruses (when tested in non-resistant rabbits) to maintain this nexus between virulence and transmission, in turn setting up an arms race between host and virus. As we describe here, this can lead to dramatic changes in the disease phenotype in non-resistant rabbits.

There is an implicit idea that changes in virulence will be due to mutations in genes involved in immunomodulation or host-range functions [40]. The role of many MYXV genes in virulence has been defined by single gene knock-out studies using the Lu strain or an early French derivative, the T1 strain [52]. In particular, the *M005L/R* and *M153R* genes have each been shown to have major virulence functions. Rabbits infected with knock-outs of either gene had a much lower CFR: 30% for Δ*M153R* and 0% for Δ*M005L/R* compared to 100% for Lu [32, 29]. However, all three Yorkshire viruses have mutations that are predicted to disrupt both these genes causing loss of key functional domains [33, 30] but have CFRs of nearly 100%. This suggests three possible explanations for retained virulence: (i) epistatic mutations compensating for the loss of these genes; (ii) a mechanism for suppressing reading frame disruptions; or (iii) functional activity retained by the truncated protein (potentially in a new role) [53]. Although it seems likely that unique amino acid substitutions are often responsible for alterations in virulence, the number of such amino acid changes evidently makes specific virulence determinants difficult to identify. Similarly, the Californian MSW strain of MYXV, which is found in *S*. *bachmani* in North America and is the most virulent strain of MYXV described for European rabbits [5, 54], has disrupted multiple virulence genes, suggesting that multiple epistatic mutations play a role in virulence determination [36].

As well as broad trends in virulence during the early radiation, changes were also observed in the clinical appearance of infected rabbits, with a relatively rapid evolution of a flat lesion morphology in both Australia and Europe rather than the domed SLS and Lu lesions [5, 15]. More recently, the amyxomatous phenotype in European isolates has been distinguished from the nodular type of disease by having few or no cutaneous lesions and, in some cases, apparently prolonged incubation periods [44, 55, 56]. For some Australian isolates the amyxomatous phenotype is seen in laboratory rabbits, although the same virus causes a nodular phenotype when tested in resistant wild rabbits suggesting that changes in the pathogenesis of the disease have occurred due to selection in resistant wild rabbits [57].

Combined, these data strongly suggest that the accumulation of mutations in field strains of MYXV has caused changes in the pathogenesis of myxomatosis, such that we now see a spectrum of disease types that depend on the interactions between the virus genome and the genetics of the rabbit and non-genetic (rabbit) factors such as microbial flora, parasites, and abiotic environmental factors including temperature [58]. As an example, field isolates of European amyxomatous viruses tested in specific pathogen-free laboratory rabbits caused relatively minor disease with few fatalities. However, the same viruses tested in rabbits from commercial rabbitries caused significant disease with severe bacterial bronchopneumonia as the most common cause of death [46, 59]. Different environmental conditions and vectors may therefore facilitate selection of virus strains that are more successful in particular niches. For example, in the farmed domestic rabbit populations in Europe where there has been no selection for resistance, we may expect low virulence strains predominantly transmitted by contact, strains with prolonged incubation periods [60, 61], or high virulence strains that can overcome imperfect vaccination [60, 56, 37].

With the exception of Yorkshire 127, rabbits that died or required euthanasia early in the course of the disease had very different clinical signs from those infected with Lu. Hemorrhage and acute pulmonary oedema were common together with high titres of virus in lungs and liver. In some cases, large numbers of coccoid bacteria were present in multiple tissues, but did not elicit a visible cellular inflammatory response. Lymphocyte depletion from lymph nodes and spleens was relatively common. Despite extremely high virus titres, there was very limited pathology in the epidermis and dermis of the primary inoculation site. This suggests an acute overwhelming of the rabbit immune response triggered by high viral titres in critical tissues. This outcome is also clearly distinct from the secondary gram negative bacterial infections (*Pasteurella multocida*, *Bordetella bronchiseptica*) described in the upper respiratory tract for rabbits infected with the progenitor viruses or the bacterial bronchopneumonia described with isolates from rabbit farms [59]. In our study, rabbits that did not die of acute disease developed more typical signs of myxomatosis, although upper respiratory tract occlusion and discharge was relatively mild, possibly reflecting the specific-pathogen free status of the rabbits.

Whether the difference in survival time and clinical disease between the acutely affected animals and the more chronically affected longer term survivors is related to genetic factors in the outbred rabbits or some stochastic factor early in the course of disease is not clear, but these animals clearly have a different form of the disease. Virulence, using the definitions of Fenner and Marshall (1957), essentially meant the AST. However, this raises the question of what virulence means in terms of *how* a strain of MYXV causes disease? Does a more virulent virus cause a different disease, or are there many pathways to death in an infected rabbit such that the phenotype seen may be due to which particular mechanism occurred in an individual rabbit. Thus, in one animal we see hemorrhage and pulmonary oedema, yet in another we see acute death without pulmonary oedema and hemorrhage, which might have developed if the animal had survived a few hours longer. It is possible that some of the longer-term survivors have a milder form of the disease at this stage and will go on to develop the more typical form of myxomatosis, and this pathway seems to predominate in attenuated viruses such as Perthshire 1527. Clearly, virulence in this case is a more nuanced concept than generally depicted in studies of its evolution.

The parallel evolution of virulence in MYXV in the Australian and British epizootics was evidently not accompanied by the acquisition of similar mutational changes. Our detailed examination of genomics and disease phenotypes of recent isolates of MYXV from the UK radiation reveals that highly virulent and highly attenuated viruses were present in the field, but that disruptions to major virulence genes were not necessarily associated with attenuation. More striking was that the disease caused by many of these viruses was clinically distinct from that caused by the progenitor Lu strain, with alterations in tissue tropism and pathogenesis in acutely affected rabbits, again demonstrating that the virus is able to explore many pathways to evolutionary success.

## Materials and methods

### Ethics statement

Sampling was performed according to field procedures approved by the Institutional Animal Care and Use Committee of The Pennsylvania State University (IACUC # 26383 and 34489). Animal experiments were conducted under protocols approved by the Institutional Animal Care and Use Committee, Pennsylvania State University (IACUC # 33615 and 42748). All animal work adhered to the guidelines laid out in the Guide for the Care and Use of Laboratory Animals. 8th ed. National Research Council of the National Academies. National Academies Press Washington DC.

### Sample collection, virus isolation and DNA preparation

The virus isolates sequenced in this study are listed in Table 1. Samples were taken from rabbits with clinical myxomatosis gathered at multiple locations on two sites, the first located in Perthshire in central-eastern Scotland, and the second in North Yorkshire, England, collected as part of other field studies [62, 63, 64, 65]. An early isolate sampled in Belfast, Northern Ireland in 1955, was also sequenced. All viruses were isolated in RK-13 cells and passaged between 1 and 3 times to prepare seed and working stocks, from which virus DNA was prepared [66]. An aliquot of virus from the DNA preparations was used for rabbit infections.

### Genome sequencing and assembly

Virus genomes were sequenced on three different platforms: the Illumina HiSeq 2000 and MiSEq, and the Ion Torrent. For the HiSeq200, template viral DNA was processed using a TruSeq DNA sample preparation kit (Illumina) to produce a multiplex library for sequencing. Briefly, extracted viral genomic DNA (gDNA) was sheared with a Covaris AFA system, creating fragments of 50 to 7,000 bp. After end-repair, purification, and 3′ adenylation, bar-coded sequencing adapters were ligated, and 400- to 500-bp fragments were purified. Fragment enrichment and clean-up were performed with AMPure XP beads. Individual library components were quantitated by quantitative PCR (qPCR), normalized, and pooled into a final sequencing library consisting of eight different viral genomes (this included seven MYXV strains that were analyzed in a separate study), which was run on an Illumina HiSeq2000 to generate 100-bp paired-end reads. For the MiSeq, libraries were produced using the Nextera XT DNA kit (Illumina). Extracted DNA samples were quantified using a Qubit fluorometer and 1ng of each sample was used as input DNA. The standard workflow was followed: duel index barcoding of the tagmented DNA was done according to the low plexity requirements and 1.8x AMPure XP beads were used to purify the library DNA. Library normalization was performed using Illumina beads. Multiplexing of the final library occurred according to Illumina recommendations. Briefly, 5 μl of each of the 14 finished, bead-normalized libraries were combined into a library pool. Next, 24 μl of this mix was transferred to a new tube containing 576 μl HT1 buffer, mixed well, and placed at 96°C for 2 minutes to denature, followed by cooling on ice for at least 5 minutes. Denatured 8pM PhiX was then combined with the denatured library pool in a total volume of 600 μl and a final concentration of 5% to produce the final sequencing pool. Sequencing was performed on an Illumina MiSeq using either 2x75bp V3 or 2X250 V2 paired-end kits, yielding approximately 14.5M paired-end reads for each run. Isolates 1527 and 2282 were sequenced on the Ion Torrent. Genomic DNA was sheared and converted into libraries with the Ion Xpress Plus fragment kit (Ion Torrent) by following the manufacturer’s instructions. Briefly, 200ng of gDNA was sheared for 20 minutes followed by purification, nick repair and adapter/barcode ligation. The DNA libraries were then size selected on the E-Gel SizeSelect (Invitrogen) platform to yield insert sizes of ~200 bp. Libraries were quantitated on the Bioanalyzer (Agilent) and combined in equimolar amounts to make the final sequencing pool. This pool was sequenced on the Ion Torrent with a 316 chip and a 200 base read length target, yielding 2.6M useable reads.

Demultiplexed reads were quality trimmed using the trim.pl perl script (http://wiki.bioinformatics.ucdavis.edu/index.php/Trim.pl) and assembled with the Velvet *de novo* assembler iterated across a range of k-mers from 45 to 65 for each assembly [67]. Contigs were ordered into a single scaffold for each genome using the Abacas.pl script [68] and the Lu genome as reference (GenBank accession AF170726), and for each assembly the k-mer that generated the most complete coverage of the reference genome was selected for finishing and downstream analysis. The quality of each scaffold was verified by remapping the untrimmed reads to the assembly using Smalt (http://www.sanger.ac.uk/science/tools/smalt-0). One region of ambiguous assembly was amplified by PCR and sequenced using Sanger methodology to confirm the assembly. A nucleotide deletion within a homopolymer run in the *M153R* gene was also confirmed by Sanger sequencing. In every case, only one complete or near complete copy of the terminal inverted repeat (TIR) was assembled at either the 5’ or the 3’ end. The Belfast 1955 isolate was assembled *de novo* on a 100K sub-sample of the cleaned, paired-end reads using CLC Genomics (version 8) with a word and bubble size of 30 nt and 150 nt, respectively. This yielded two contigs corresponding to the core genome (~138K) and TIR (~11K). The TIR contig was duplicated and reverse complemented before manually assembling onto the core genome, and then all the cleaned, paired-end data was re-mapped back to confirm final assembly.

Genome annotation was transferred from the Lu strain to the newly sequenced MYXV genomes using the Rapid Annotation Transfer Tool [69]. EMBL flatfiles of transferred gene models were then inspected and compared to the Lu reference using the Artemis Comparison Tool [70]; incorrect models were corrected, and new gene models added where transfer had not occurred.

Nucleotide sequence accession numbers: all genome sequences generated here have been deposited in GenBank (https://www.ncbi.nlm.nih.gov/) under accession numbers KY548792-KY548813 (S10 Table).

### Evolutionary analysis

The 22 MYXV genome sequences determined here were combined with 35 complete genomes available on GenBank, representing 25 from the Australian outbreak (including the SLS release strain) and 10 from Europe (including the Lu release strain) (S10 Table). These sequences were initially aligned in MUSCLE [71] and adjusted manually, resulting in a final sequence alignment data set of 57 sequences 163,645 bp in length. Because the sequences are highly conserved, the locations of synonymous and non-synonymous mutations in these sequences were determined manually.

An initial phylogenetic tree of these sequences was inferred using the maximum likelihood procedure available in the PhyML package [72]. This analysis utilized the HKY+Γ_4_ model of nucleotide substitution and NNI+SPR branch-swapping. To test for the presence of recombination we utilized the RDP, Genecov and Bootscan methods (with default settings) available within the RDP4 package [73]. No significant evidence for recombination was found.

To determine the rate of MYXV evolution we first assessed the degree of clock-like structure in the data using a regression of root-to-tip genetic distances on the ML tree inferred above against the year of virus sampling using TempEst [74]. As this analysis revealed strong temporal structure (see Results), we next inferred the rates and dates of viral evolution using the Bayesian Markov chain Monte Carlo (MCMC) approach available in the BEAST package [75]. For this analysis we used a range of nucleotide substitution (HKY+Γ_4_, GTR+Γ_4_), molecular clock (strict, relaxed uncorrelated lognormal) and demographic (constant, Bayesian skyride) models. As these gave strongly overlapping results we based our analysis on the simplest model: HKY+Γ_4_, strict clock, constant population size (Fig 1). All analyses were run twice and for sufficient time (100 million generations) to ensure that convergence was achieved, with statistical uncertainly manifest in values of the 95% highest posterior distribution (HPD). The posterior distribution of trees from the HKY+Γ_4_, strict clock, constant population size run was also used to infer a maximum clade credibility (MCC) tree (Fig 1). The degree of support of individual nodes is depicted as posterior probability values.

### Animal studies

New Zealand White male laboratory rabbits (*Oryctolagus cuniculus*) of four months of age were purchased from Harlan Laboratories (Oakwood facility). Rabbits were specific-pathogen-free for *Pasteurella multocida* and *Bordetella bronchiseptica*. Animals were housed in individual cages on a 12h light regime, fed 125 g of standard pellets per day and allowed 10 days to acclimate in the facility prior to infection.

Groups of six rabbits were inoculated with 100 pfu of virus intradermally in the rump and monitored closely over the course of the infection. Daily clinical examination included: rectal temperature, body weight, primary lesion size and shape at the inoculation site, secondary lesion size and distribution, plus semi-quantitative scoring on a 0 to 3 scale for demeanour, eyelid swelling, ear swelling, anogenital swelling, scrotal oedema, blepharoconjunctivitis, nasal discharge and respiratory difficulty. Food and water intake were recorded and fecal and urinary output monitored by inspection of collecting trays under the cages. Rabbits were euthanized based on the degree of clinical severity using respiratory difficulty, depression, inanition, reluctance to move, weakness on handling, weight loss and failure to eat or drink as indicators; any rabbit exhibiting pain or with a subnormal temperature was immediately euthanized.

To monitor virus replication at the primary inoculation site, 1 mm diameter dermal punch biopsies were collected: in each group, three rabbits were sampled at day 5 post-infection and three at day 7; thereafter, each surviving rabbit was sampled at 5 day intervals. DNA was prepared using the DNeasy kit (Qiagen).

Rabbits were autopsied as soon as possible after death and bodies refrigerated if autopsy was delayed. Samples of the primary lesion and other tissues were collected for virus titration and histology but only from euthanized rabbits or rabbits that died within 1–2 hours prior to autopsy. Blood samples were collected from the marginal ear vein at days 0 and 10, or following euthanasia, by cardiac puncture. Hematology was performed by the Centralised Biological Laboratory Facilities, the Pennsylvania State University.

### Challenge infections with Yorkshire 135 in immune rabbits

Because of the unusual virulence of the Yorkshire 135 virus, we tested whether there was any adventitious agent in the virus preparation by challenging immune rabbits with Yorkshire 135. The only reaction was a swelling at the inoculation site, which resolved by day 6. This is typical of what is seen when immune rabbits are challenged. While this does not completely exclude an adventitious agent that was only pathologic in the context of a highly immunosuppressive MYXV infection, it strongly supports the hypothesis that the peracute disease seen with Yorkshire 135 was indeed due to MYXV.

### Survival analysis

To enable comparison with previous studies of MYXV, survival times (ST) from inoculation to death were estimated for rabbits that were euthanized as follows: (i) moribund rabbits were assigned the time of euthanasia as the ST; (ii) rabbits that were not expected to survive the next 24 hours were assigned an additional ST of +12 hours; and (iii) rabbits euthanized for humanitarian reasons were assigned a ST of +48 hours. Animals found dead were assigned a ST half-way between the time of last observation and finding the body. Average survival times (AST) for each group were calculated from individual ST normalized using the procedure of Fenner and Marshall (1957) [5] as log_10_(ST-8) and then back-transformed; a survival time of 60 days was assigned to rabbits that recovered or were alive at the end of the trials and considered likely to recover. If more than two rabbits survived, the virulence grade was assigned based on the CFR and clinical severity. Virulence grades were based on Fenner and Marshall (1957) [5] as modified by Fenner and Woodroofe (1965) [43] (Table 4). Data were also analysed using Kaplan-Meier survival plots (using actual inferred survival times rather than the normalized survival times) and tested for statistical significance by log rank test implemented in SigmaPlot.

**Table 4:** MYXV virulence classification.

	^1^Grade 1	Grade 2	Grade 3A	Grade 3B	Grade 4	Grade 5
CFR	>99	95–99	90–95	70–90	50–70	<50
AST	≤ 13	14–16	17–22	23–28	29–50	n/a

### Real-time PCR

Quantitative PCR (qPCR) was performed on an ABI 7500-fast machine, using the Quantifast Sybr green kit (Qiagen), by amplification of a 126 bp fragment (nt 584–710) from the *M080R* gene from DNA extracted from primary lesion biopsies. This was quantified on a standard curve using a linearized control plasmid containing a 642 bp region of the *M080R* gene (nt 241–883). None of the UK viruses have mutations in this sequence. Virus titres were expressed as genome copy number/mg tissue. The qPCR primers used were: M080 qPCR Forward: 5' TATCAAACAACCTCCGCATACC 3' (*M080R* 584–605) and M080 qPCR Reverse: 5' CTCCCATAACGCTTCCGAC 3' (*M080R* 710–692)

### Plaque assays

Samples of the primary lesion, lung, liver, spleen, and right popliteal lymph node were collected at autopsy from euthanized rabbits. Tissues were homogenized by Tissuelyser (Qiagen). Virus was titrated on RK-13 cell monolayers as previously described [49] with titres expressed as pfu/g of tissue.

## Supporting information

S1 Fig The Yorkshire M036 mutation. Codon alignment of *M036L* from codons 432 to 441. Sussex has an A insert at nt 1300 in *M036L*, which disrupts the ORF. The Yorkshire lineage viruses, which are phylogenetically related to Sussex, all have the A at 1300 but either have had a G deletion at 1303 or have had two mutations in codon 434 (shown in bold below the first York sequence). This corrects the reading frame and restores the correct amino acid sequence with an M434N mutation at codon 435. The lineage 1 Perthshire viruses (1527) and lineage 2 Perthshire viruses (2082) have an AT deletion after 1303 and 1304 respectively. The Perthshire lineage 2 viruses also have an earlier indel at nt 973.
(DOCX)

S2 Fig Virus loads in primary lesions. Copy number /mg of tissue of a segment of the M080R gene measured by quantitative PCR on biopsies of primary lesions at staggered 5 day intervals from day 5; 3 rabbits per time point were biopsied at 5, 7, 10, 12, 15, 17, 20, 22 days. At later time points not all animals could be sampled due to deaths or euthanasia.
(PDF)

S1 Table (A) Genes with no mutations in both the UK and Australian MYXV isolates. (B) Genes with only synonymous mutations in both the UK and Australian viruses.
(DOCX)

S2 Table Indels in genes that do not disrupt the ORF. (DOCX)

S3 Table Clinical time course of UK virus isolates and Lausanne. (DOCX)

S4 Table Clinical phenotypes of Lausanne and modern UK viruses. (DOCX)

S5 Table Pathology of Lausanne and modern UK viruses. (DOCX)

S6 Table (A). Virus titres in Lausanne infected rabbits at day 12 after infection. (B). Virus titres in tissues at autopsy for UK modern isolates.
(DOCX)

S7 Table Amino acid differences between the Yorkshire lineage viruses. (DOCX)

S8 Table Amino acid differences between the Perthshire lineage 1 viruses. (DOCX)

S9 Table Amino acid differences between the Perthshire lineage 2 viruses. (DOCX)

S10 Table All the MYXV sequences used in the evolutionary analysis. (DOCX)
