# Supplementary material for: Genomic and phenotypic characterization of myxoma virus from Great Britain reveals multiple evolutionary pathways distinct from those in Australia
Source: PLoS Pathog. 2017 Mar 2;13(3):e1006252. doi: 10.1371/journal.ppat.1006252 (PMC5349684; doi:10.1371/journal.ppat.1006252)
Supplement: S1 Fig — Codon alignment of M036L from codons 432 to 441. Sussex has an A insert at nt 1300 in M036L, which disrupts the ORF. The Yorkshire lineage viruses, which are phylogenetically related to Sussex, all have the A at 1300 but either have had a G deletion at 1303 or have had two mutations in codon 434 (shown in bold below the first York sequence). This corrects the reading frame and restores the correct amino acid sequence with an M434N mutation at codon 435. The lineage 1 Perthshire viruses (1527) and lineage 2 Perthshire viruses (2082) have an AT deletion after 1303 and 1304 respectively. The Perthshire lineage 2 viruses also have an earlier indel at nt 973. (DOCX) [file ppat.1006252.s001.docx]

**S1 Fig.** The Yorkshire M036 mutation.

*M036L*: codon 432 to 441

Lu ATC ATC - ATG GAT ACG ATG GAT CAT ATC ATG

Sussex ATC ATC A ATG GAT ACG ATG GAT CAT ATC ATG

York ATC ATC A AT- GAT ACG ATG GAT CAT ATC ATG

ATC ATC A**AT** GAT ACG ATG GAT CAT ATC ATG

1527 ATC ATC - ATG G-- ACG ATG GAT CAT ATC ATG

2082 ATC ATC - ATG G-- ACG ATG GAT CAT ATC ATG

Codon alignment of *M036L* from codons 432 to 441. Sussex has an A insert at nt 1300 in *M036L*, which disrupts the ORF. The Yorkshire lineage viruses, which are phylogenetically related to Sussex, all have the A at 1300 but either have had a G deletion at 1303 or have had two mutations in codon 434 (shown in bold below the first York sequence). This corrects the reading frame and restores the correct amino acid sequence with an M434N mutation at codon 435. The lineage 1 Perthshire viruses (1527) and lineage 2 Perthshire viruses (2082) have an AT deletion after 1303 and 1304 respectively. The Perthshire lineage 2 viruses also have an earlier indel at nt 973.
