# Supplementary figures and images for: Genomic and phenotypic characterization of myxoma virus from Great Britain reveals multiple evolutionary pathways distinct from those in Australia
Source: PLoS Pathog. 2017 Mar 2;13(3):e1006252. doi: 10.1371/journal.ppat.1006252 (PMC5349684; doi:10.1371/journal.ppat.1006252)

Perthshire 1527

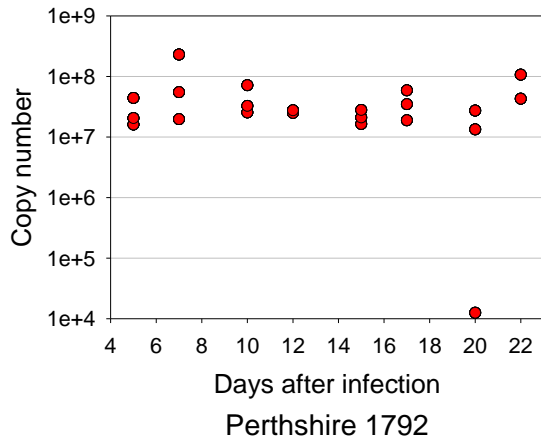

Perthshire 1537

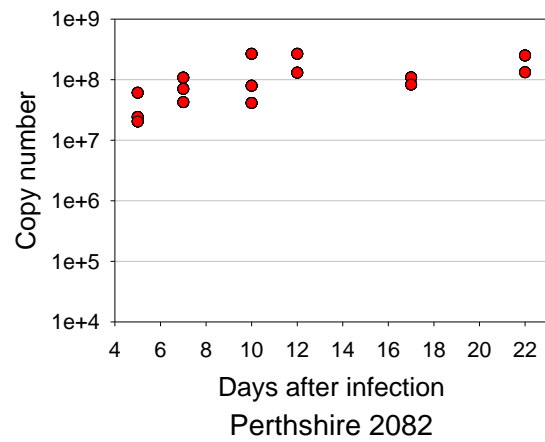

Perthshire 1792

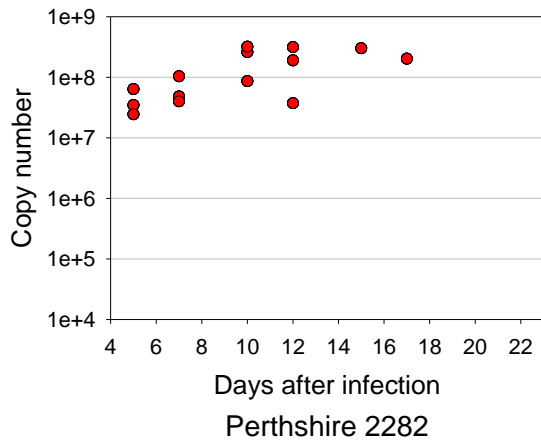

Perthshire 2082

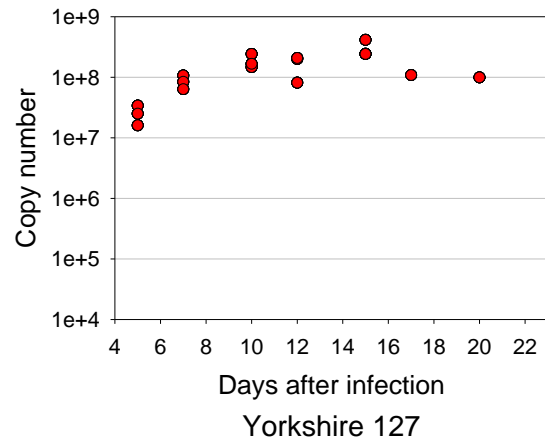

Perthshire 2282

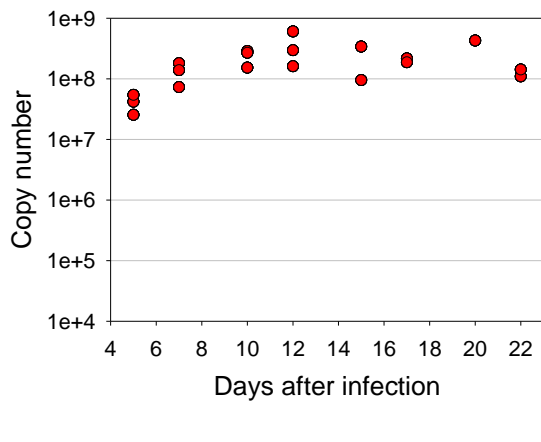

Yorkshire 127

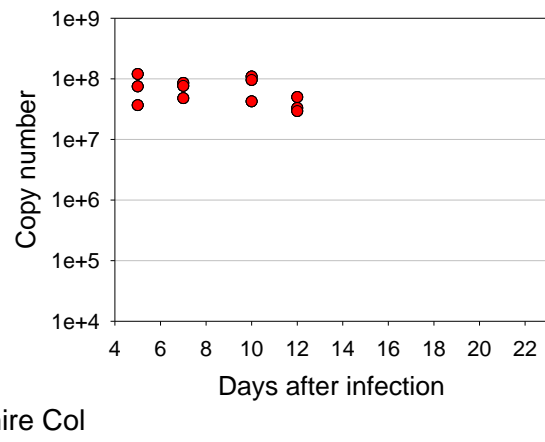

Yorkshire Col

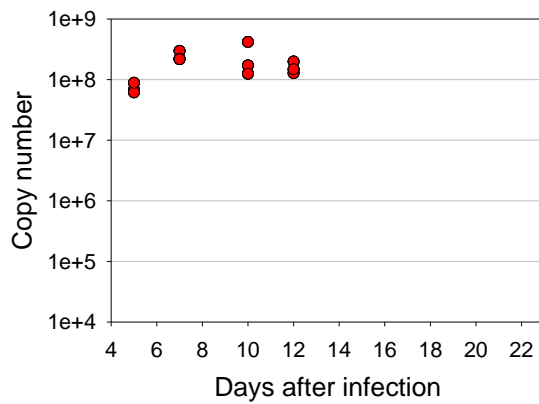

Supplement: S2 Fig — Copy number /mg of tissue of a segment of the M080R gene measured by quantitative PCR on biopsies of primary lesions at staggered 5 day intervals from day 5; 3 rabbits per time point were biopsied at 5, 7, 10, 12, 15, 17, 20, 22 days. At later time points not all animals could be sampled due to deaths or euthanasia. (PDF) [file ppat.1006252.s002.pdf]
