# Supplementary material for: Genomic and phenotypic characterization of myxoma virus from Great Britain reveals multiple evolutionary pathways distinct from those in Australia
Source: PLoS Pathog. 2017 Mar 2;13(3):e1006252. doi: 10.1371/journal.ppat.1006252 (PMC5349684; doi:10.1371/journal.ppat.1006252)
Supplement: S1 Table — (A) Genes with no mutations in both the UK and Australian MYXV isolates. (B) Genes with only synonymous mutations in both the UK and Australian viruses. (DOCX) [file ppat.1006252.s003.docx]

**S1 Table.** A. Genes with no mutations in both the Australian and UK MYXV isolates.

| **Gene** | **Length (nt)** | **Function** | **Function class** |
| --- | --- | --- | --- |
| *M011L* | 498 | antiapoptosis; lymphocyte host-range | host-range/immunomodulatory |
| *M024L* | 444 | unknown | unknown |
| *M035L* | 288 | thiol oxido-reduction (VACV *E10L*) | assembly S-S bond formation |
| *M037L* | 96 | entry fusion complex (VACV *03L*) | fusion/entry |
| *M039L* | 222 | membrane protein | structural |
| *M050R* | 189 | RNA pol subunit rpo 7 | gene transcription/DNA metabolism |
| *M055R* | 726 | mature virion membrane protein | structural |
| *M059R* | 387 | entry fusion complex | fusion/entry |
| *M069L* | 534 | Thr/Ser phosphatase | core enzyme |
| *M074R* | 945 | DNA topoisomerase 1 | gene transcription/DNA metabolism |
| *M075R* | 441 | unknown; VACV H7R | unknown |
| *M077L* | 429 | core 7 protein assembly complex | structural |
| *M082R* | 489 | RNA pol subunit rpo 18 | gene transcription/DNA metabolism |
| *M089L* | 447 | late transcription factor-2 | gene transcription/DNA metabolism |
| *M091L* | 225 | thiol oxido-reduction (VACV *A2.5L*) | assembly S-S bond formation |
| *M095L* | 1119 | core protein ( VACV *A6L*) | structural |
| *M098L* | 228 | membrane protein | structural |
| *M100R* | 939 | scaffold protein | structural |
| *M102L* | 204 | membrane protein; DNA encapsidation | structural |
| *M104L* | 159 | potential immunomod/unknown | host-range/immunomodulatory |
| *M117L* | 906 | RNA pol subunit rpo 35 | gene transcription/DNA metabolism |
| *M118L* | 228 | core 7 protein assembly complex | structural |
| *M131R* | 489 | super oxide dismutase inhibition | host-range/immunomodulatory |

B. Genes with only synonymous mutations in both the UK and Australian viruses.

| **Gene** | **Length (nt)** | **Function** | **Function class** |
| --- | --- | --- | --- |
| *M019L* | 645 | entry fusion complex | fusion/entry |
| *M022L* | 1113 | extracellular virion prot (VACV *F13L*) | structural |
| *M026R* | 306 | DNA binding phosphoprotein | structural |
| *M030L* | 666 | RNA pol subunit/ intermediate transcription factor-1 | gene transcription/DNA metabolism |
| *M033R* | 816 | core protein | structural |
| *M051R* | 522 | unknown (VACV *G6R*) | unknown |
| *M053R* | 780 | late transcription factor-1 | gene transcription/DNA metabolism |
| *M056R* | 297 | unknown (VACV *L2R*) | unknown |
| *M060R* | 444 | core 7 protein assembly complex | structural |
| *M066R* | 555 | RNA pol subunit rpo 22 | gene transcription/DNA metabolism |
| *M073R* | 582 | late transcription factor-4 | gene transcription/DNA metabolism |
| *M079R* | 654 | uracil deglycosylase | gene transcription/DNA metabolism |
| *M080R* | 2358 | nucleoside triphosphatase | gene transcription/DNA metabolism |
| *M086L* | 1896 | nucleoside triphosphatase 1/ DNA helicase | gene transcription/DNA metabolism |
| *M088L* | 1622 | mature virion scaffold protein | structural |
| *M090L* | 672 | late transcription factor-3 | gene transcription/DNA metabolism |
| *M101L* | 483 | core protein | structural |
| *M105L* | 282 | core 7 protein assembly complex | structural |
| *M113R* | 1155 | Intermediate transcription factor-3 | gene transcription/DNA metabolism |
| *M119L* | 150 | unknown | unknown |
| *M120L* | 765 | ATPase; DNA encapsidation | enzyme |
| *M123R* | 537 | VACV *A35R* precursor | structural |
| *M133R* | 1689 | DNA ligase | gene transcription/DNA metabolism |
