# Supplementary material for: Genomic and phenotypic characterization of myxoma virus from Great Britain reveals multiple evolutionary pathways distinct from those in Australia
Source: PLoS Pathog. 2017 Mar 2;13(3):e1006252. doi: 10.1371/journal.ppat.1006252 (PMC5349684; doi:10.1371/journal.ppat.1006252)
Supplement: S2 Table — (DOCX) [file ppat.1006252.s004.docx]

**S2 Table.** Indels in genes that do not disrupt the ORF.

| **Gene** | **Indel** | **Protein function** | **Virus** |
| --- | --- | --- | --- |
| *M009L* | 9 nt del (but ORF already disrupted) | Predicted E3 Ub ligase | Perthshire 1812 |
| *M013L* | 12 nt insertion; KAVT repeat sequence | PYD domain; inhibition of caspase 1 activation & NFκB | Yorkshire lineage |
| *M063R* | 15 nt del; TEEED direct repeat deleted at C-terminus | Host-range; essential for replication in rabbit cells | Perthshire lineage 2 |
| *M093L* | 27 nt del; aa 92-100 | Core protein; VACV A4L | Yorkshire lineage |
| *M125R* | 18 nt del; aa 134-139 | Unknown | Perthshire lineage 2 |
| *M134R* | 45 nt del; aa 640-654; 12 of which are a direct repeat | Unknown/structural? | Perthshire lineage 1 |
