# Supplementary material for: Genomic and phenotypic characterization of myxoma virus from Great Britain reveals multiple evolutionary pathways distinct from those in Australia
Source: PLoS Pathog. 2017 Mar 2;13(3):e1006252. doi: 10.1371/journal.ppat.1006252 (PMC5349684; doi:10.1371/journal.ppat.1006252)
Supplement: S3 Table — (DOCX) [file ppat.1006252.s005.docx]

**S3 Table**. Clinical time course of UK virus isolates and Lausanne.

| **Day** | **Lausanne** | **Perthshire 2082** | **Perthshire 2282** | **Perthshire 1792** | **York Col** | **York 135** | **York 127** | **Perthshire 1527** | **Perthshire 1537** |
| --- | --- | --- | --- | --- | --- | --- | --- | --- | --- |
| 3 | All + at inoculation site | + or +/- at inoculation site | All + at inoculation site | 5/6 + or +/- at inoculation site | All + or +/- at inoculation site | 4/6 + at inoculation site | All + or +/- at inoculation site | All + or +/- at inoculation site | 5/6 + or +/- at inoculation site |
| 5 | Primaries 1.7-2.3 cm diameter; slight swelling or red spots on eyelid margins; slight swelling base of ears; 2/6 febrile | Primaries 1.2-1.6 cm diameter; red spots on eyelid margins | Primaries 1.5-2cm; slight swelling of eyelids, ears, heads | Primaries 1-2.4 cm; early slight swelling of face, ears, eyelids | Primaries 1.6-2.5 cm; slight swelling of ears; redness of eyelid margins; 1/6 slight anogenital swelling | Primaries red spot to 1.4 cm; all have redness/swelling of eyelid margins; 3/6 have slight swelling at base of ears | Primaries 1.5-2 cm diameter, flat to slightly raised; slight swelling or red spots on eyelid margins; slightly swollen heads | Primaries 1.3-2.2 cm diameter, raised; slight swelling at base of ears, eyelids and head; 4/6 febrile. | Primaries 1.1-2.5 cm, slightly raised; slight swelling of base of ears, eyelids and head; 4/6 febrile |
| 7 | Much quieter; eyelids swollen; heads swollen; increasing anogenital swelling; ears swollen at base | Eyelid and ear swelling; slight anogenital swelling; primaries 2.2-2.7 cm | Slight swelling of ears, eyelids and genital pore; primaries 1.9-2.9cm; one has purple rash on ear | Slight eyelid and ear swelling; primaries 1.8-3cm | Eyelids and ears mild to moderate swelling (1-2); slight anogenital swelling; all have swollen heads and one is losing hair; primaries 2.1-3cm | All have swollen heads; eyelids slight to mild swelling; ears slightly to mildly swollen; some anogenital reaction; food consumption very poor | Primaries 2.3-3 cm, slightly raised – dark crimson with black centres; mild eyelid and ear swelling; slight to mild anogenital swelling | Primaries 1.9-3cm raised, crimson; slightly swollen ears, eyelid margins slightly swollen or red; slight anogenital swelling; 5/6 febrile; secondaries developing | Primaries 2-3 cm, raised, crimson; slight swelling of ears and eyelids or above eyelids and slightly swollen heads; food consumption down |
| 10 | Very quiet to depressed; primaries 2.8-3.8 cm diameter & scabbing or oozing. Eyelids grossly thickened, copious white discharge; severe anogenital swelling; very swollen lips; secondaries on head, legs, body | Eyelids swollen partially closed; swollen heads and ears; primaries 2.3-3.2 cm; slight anogenital swelling | Eyelids swollen partially closed; ears moderately swollen; heads swollen – some hair loss; mild to moderate anogenital swelling | Eyelids almost fully closed and ears swollen; slight anogenital swelling; several have swollen heads; food consumption reduced; primaries 2-3.2 cm | Eyelids very swollen 2/3 to fully closed; all have some discharge; very swollen heads with hair loss; ears very swollen; moderate to severe anogenital swelling; food intake very reduced; primaries 2.3-3.2 cm slightly raised or flat | 3/6 surviving; eyelids 2/3 to fully closed; one has blood on ear; one has very swollen ears; not eating. Primaries small ≤1.6 cm. | Primaries 2.4-4.2 cm, raised, fleshy, flat, purple/black; very swollen eyelids with considerable mucoid discharge; ears moderately to very swollen and becoming purple; heads very swollen; some nasal obstruction; scrotal swelling or orchitis /epididymitis; depression; poor food intake | Primaries 2-4 cm raised often demarcated at the edges, crimson/purple; secondary lesions on ears and eyelids; several have swollen heads; most eating well; 5/6 febrile; one rabbit more severely affected | Primaries 2.3-3.2 cm, flat to raised, crimson/pink; eyelids 1/2 to 2/3 closed; ears swollen starting to droop; all have slight to moderate anogenital swelling; very swollen heads; food consumption poor |
| 12 | Miserable appearance; extremely swollen, closed eyelids; extreme anogenital swelling; wt loss; temperatures decreased to sub normal; some have respiratory difficulty; primaries 3.1-4 cm diameter. All euthanized or died d12. | Eyelids 2/3 to fully closed; ears very swollen; increasing anogenital swelling; food consumption down; slight scrotal swelling in one | Eyelids moderately to severely swollen; heads swollen with hair loss; mild to moderate anogenital swelling; one not eating | Eyelids half to 2/3 closed; ears moderately swollen; 4/6 have swollen heads; mild anogenital swelling; food intake poor; one euthanized – respiratory difficulty | Eyelids 2.5 to 3 – very swollen to completely closed – lot of mucoid discharge; ears very swollen; anogenital swelling is slight to severe; 2/6 some scrotal swelling; primaries 2.2-2.7 cm; one euthanized | 1/6 surviving; eyelids swollen closed with some discharge; ears moderately swollen; slight anogenital swelling; depressed. Died day 12. | 5/6 surviving; all depressed with 3/5 exhibiting some respiratory difficulty due to nasal obstruction; primaries raised 2.8-3.6 cm, crimson/purple; one is demarcating (this rabbit will recover); eyelids fully closed except in one rabbit; ears very swollen; ears and eyelids purple; moderately severe anogenital swelling; poor food intake | Primaries 3-4 cm, raised, purple/black demarcated from surrounding tissue; all have secondaries; eyelid swelling very mixed – some rabbits very mild disease but 2 more severely affected with anogenital swelling and scrotal swelling and eyelids 2/3 closed | 5/6 surviving; primaries 2.3-3.2 cm, slightly raised, pink/red; eyelids 2/3 to fully closed with discharge; ears swollen; one has secondaries on ears and scrotal swelling (will go on to survive). |
| 15 |  | 5/6 surviving; Some ocular discharge and nasal noise; mod to severe anogenital swelling; some scrotal swelling (3/5); primaries 2-3 cm; depressed; not eating; one bleeding from ear | 4/6 surviving; primaries 2.5-2.9 cm; eyelids 2/3 to completely closed with some discharge; some nasal obstruction; moderate to severe anogenital swelling in 3/4; all still eating | 3/6 surviving; eyelids 2/3 to fully closed; ears moderately to severely swollen; mild anogenital swelling; 1/3 slight scrotal swelling; depressed; little food intake | 3/6 surviving; eyelids fully closed with copious mucoid discharge; some respiratory obstruction and difficulty; scrotal swelling or orchitis /epididymitis |  | 1/6 surviving; secondary lesions on ears, face, eyelids; ears are purple and scabbing | 6/6 surviving; primaries 2.7-4cm, raised, crimson/black and scabbing; only 2 have closed or partially closed eyelids and both these are depressed; others are bright and alert | 2/6 surviving; primaries 3 and 3.6 cm, raised and fleshy; eyelids swollen 2/3 closed + discharge; anogenital swelling and scrotal swelling; secondary lesions developing; some nasal obstruction |
| 17 |  | 4/6 surviving; ears very swollen; eyelids 2/3 to fully closed with some discharge; 2/4 scrotal swelling; some nasal obstruction; depressed & inappetant | 4/6 surviving; 3/4 have swollen closed eyelids; increasing depression but still eating; some nasal obstruction and increasing anogenital swelling | 2/6 surviving; fully closed eyelids with discharge; nasal obstruction; poor to no food intake | 1/6 surviving; depressed but eating and drinking and moving around; lot of nasal and ocular discharge; scrotal oedema |  | Slow laboured respiration; primary raised/fleshy; resolving scrotal oedema; purple/black haemorrhages over ears; eating, drinking, grooming. | Primaries 2.4-3.7 cm raised, demarcated and scabbing except for the 2 severely affected rabbits – still crimson and fleshy; these 2 also have swollen ears and eyelids and ocular discharge; one is not eating. | Not a lot of change from d15. |
| 20 |  | Single survivor euthanized: swollen eyelids – mucoid/white discharge; copious mucopurulent nasal discharge; swollen purple ears; severe anogenital and scrotal swelling; still eating and drinking a little but very weak | 3/6 surviving; large clinical decline after day 17; soiling of perineum; completely closed eyelids; very swollen ears; depression; still eating | Last survivor euthanized; depressed and struggling to breathe due to upper respiratory tract obstruction; still eating and drinking a little | Last survivor was euthanized at day 18 |  | Primary and secondaries are scabbing; ocular discharge is reduced; breathing easier; eaten all its ration | Euthanized one of the severely affected rabbits because of wt loss; one other is still severely affected but eating. One of the others has severe anogenital swelling | Both have very swollen heads and ears; eyelids never fully closed but becoming distorted; considerable nasal discharge, crusting and obstruction; respiration slow and somewhat laboured; one rabbit is improving a little. |
| 22 |  |  | 2/3 euthanized – resp difficulty; wt loss; one survivor was euthanized d25 |  |  |  | Continuing to eat well and groom; secondaries now on feet but secondaries are scabbing; still has fever. | Only one rabbit is not starting to recover and it is starting to eat reasonably. | Food consumption very poor; both have a lot of nasal discharge and obstruction. |
