# Supplementary material for: Genomic and phenotypic characterization of myxoma virus from Great Britain reveals multiple evolutionary pathways distinct from those in Australia
Source: PLoS Pathog. 2017 Mar 2;13(3):e1006252. doi: 10.1371/journal.ppat.1006252 (PMC5349684; doi:10.1371/journal.ppat.1006252)
Supplement: S4 Table — (DOCX) [file ppat.1006252.s006.docx]

**S4 Table**. Clinical phenotypes of Lausanne and modern UK viruses.

| **Virus** | **Clinical disease** |
| --- | --- |
| Lausanne progenitor virus: Grade 1 virulence | 1.2-2 cm red swelling at inoculation site by d3; temperature rise from d5; distinctly quieter by d7 with swelling of heads, eyelids, ano-genital region and base of the ears. By d10 there was a marked worsening of clinical signs with eyelids up to 1 cm thick, primary lesions 3-4 cm in diameter often scabbing or oozing blood; secondary lesions on head, body and legs; severely swollen lips and severe anogenital swelling. At d12, there was significant wt loss, depression, decreased temperature and respiratory difficulty in some cases. All euthanized or died at d12. |
| 1527 Perthshire  lineage 1:  Grade 5 virulence | Large range of clinical signs from limited disease with rapid recovery to quite severe myxomatosis in 2 rabbits. Reaction at primary site seen at days 2-3; Temperature rise from d5. Primaries large (3-4 cm diameter by d15), raised, flat and demarcated from surrounding tissue; secondaries on ears, face, eyelids, legs. One rabbit euthanized at d20 for inappetance/wt loss but may have recovered. |
| 1537  Perthshire  lineage 1:  Grade 3/4 virulence | Reaction at inoculation site by d3. Temperature rise from d4; very swollen heads developing from d7; large, raised, red primary lesions by d10. 4 acute deaths between d11-14 with relatively minor clinical signs; remaining 2 developed typical myxomatosis with secondary lesions, blepharoconjunctivitis with swollen distorted eyelids and nasal discharge and crusting but primaries did not scab. At least one may have recovered. |
| 1792  Perthshire  lineage 1:  Grade 2 virulence | Primaries poorly differentiated from surrounding tissue; no secondary lesions; swollen heads; biphasic temperature response from d4. Marked reduction in food intake by d10. All dead/euthanized between d12-20. Tendency to bleed in later stages; depression and respiratory difficulty. |
| 2282  Perthshire  lineage 2:  Grade 3 virulence | Early reaction at inoculation site (d2); biphasic temperature rise from d6; swollen heads from d8; primaries 2.5-3.6 cm diameter at d10; slightly raised. Two early acute deaths at d13 &14; rabbits euthanized later had fairly typical myxomatosis but secondaries were rare. Transient weight gain in rabbits that survived longer probably due to oedema. Final rabbit may have recovered but was euthanized on humanitarian grounds. |
| 2082  Perthshire  Lineage 2:  Grade 3 virulence | Slowly developing with very swollen eyelids and ears; primaries poorly differentiated from surrounding tissue, flat to slightly raised; no or very few discrete secondary lesions; tendency to bleed from nose, ears and feet during acute phase. Biphasic temperature response from d6. |
| Yorkshire 127:  Grade 2/3 virulence | All positive at inoculation site by d3. Temperatures elevated from d4 with slight reaction of eyelids, head and ears detectable at d5. D10: primaries raised, flat on the surface, purple/black colour 2.2-4.4cm diameter; most have eyelids swollen closed with considerable mucoid discharge; head and ears moderately to severely swollen; ears purple; some nasal discharge and scrotal swelling; secondaries developed over the next few days. 5/6 rabbits euthanized between d11-d13; the remaining rabbit recovered despite severe clinical signs. |
| Yorkshire Col:  Grade 2 virulence | Fairly rapid development of head, ear and eyelid swelling but no temperature increase until d9. All euthanized or died between d12-18: very swollen heads, eyelids completely closed with copious yellow discharge, nasal discharge, very swollen ears and ano-genital swelling; scrotal oedema/orchitis/epididymitis. No discrete secondary lesions. |
| Yorkshire 135:  Grade 1 virulence | All died or were euthanized between d7-13. Only the longest survivor developed fully closed swollen eyelids and some nasal discharge although all developed some head swelling. Hypersensitive to handling with limb spasm and rigidity in terminal stages. Primary lesions poorly differentiated from surrounding tissue. Temperatures not elevated until d7. Inappetance from d4 and wt loss; no secondary lesions. |
