# Supplementary material for: Genomic and phenotypic characterization of myxoma virus from Great Britain reveals multiple evolutionary pathways distinct from those in Australia
Source: PLoS Pathog. 2017 Mar 2;13(3):e1006252. doi: 10.1371/journal.ppat.1006252 (PMC5349684; doi:10.1371/journal.ppat.1006252)
Supplement: S5 Table — (DOCX) [file ppat.1006252.s007.docx]

**S5 Table**. Pathology of Lausanne and modern UK viruses.

| **Virus** | **Pathology** |
| --- | --- |
| Lausanne progenitor virus:  Grade 1 virulence | Extremely thickened eyelids; swollen lips and nose; base of ears swollen; extreme anogenital swelling. Popliteal lymph nodes enlarged; scrotal oedema in some cases and enlarged tail of epididymis with testes congested with blood. Superficial white scarring on the liver in some animals. Some had patchy red/crimson areas in the lungs. Primaries 4-5 cm in diameter, mostly scabbed on the surface with a necrotic red/black appearance on cut section 1.5 - 2 cm thick. 1-2 cm secondaries over body, head and legs but not pinnae of ears. Histology: lymph nodes partially depleted; neutrophil influx in primary and secondary lesions, eyelids, lymph nodes and testes. Patchy interstitial pneumonia in some animals. |
| 1527 Perthshire  lineage 1:  Grade 5 virulence | Rabbit autopsied at d20 had very enlarged lymph nodes and spleen and atrophied testes. At day 24, all rabbits had very enlarged lymph nodes and spleens; 3 had markedly atrophied testes with granulomata in the epididymides. |
| 1537  Perthshire  lineage 1:  Grade 3/4 virulence | 4 acute deaths: swollen heads and some swelling of eyelids and base of ears with purple bruising around ears; enlarged popliteal LNs and spleens; firm, swollen livers with white scarring and granular consistency; subcutaneous oedema ventral neck and front legs (probably draining from head); one had secondary lesions on ears; one animal had small haemorrhages in lungs. Histology: patchy hepatic necrosis; some inflammatory cells; bacteria present in liver sections from one rabbit. Two animals autopsied at d26 had raised red primaries 4 cm diameter; enlarged spleens and slightly enlarged LNs and atrophied testes; one had some consolidation in R lung. |
| 1792  Perthshire  lineage 1:  Grade 2 virulence | Pulmonary oedema in 4/6; lymphocytes depleted from LNs and spleen; earliest death (d12) had small numbers of bacteria in pop LN and lung with no inflammatory cells but no bacteria seen in other rabbits. Primary lesion: extensive hypertrophy, hyperplasia and degeneration of epidermal cells; at late timepoint (d20) some scabbing and acute inflammation. |
| 2282  Perthshire  lineage 2:  Grade 3 virulence | Acute deaths: pulmonary oedema, muscle necrosis/haemorrhage, massive numbers of bacteria throughout tissues but no inflammatory cells. Lymphoid tissues depleted. One animal developed an intestinal intussusception (d18). Later deaths did not have bacteria in tissues but had evidence of inflammation eg neutrophils in subcapsular sinus of LN and in eyelid sections; testes atrophied. Primary lesion: hypertrophy, hyperplasia and degeneration of epidermis with some underlying inflammation; superficial scabbing late. |
| 2082  Perthshire  Lineage 2:  Grade 3 virulence | Pulmonary oedema and haemorrhage in early 4 deaths; bacteria in lung, liver, spleen, lymph node, kidney, epididymis, heart in some rabbits. Depletion of lymphoid tissues. Animals that survived for longer have more cellular inflammatory responses. Primary lesions: hypertrophy, hyperplasia, degeneration of epidermal cells with some disruption of dermis but little inflammatory response. |
| Yorkshire 127:  Grade 2/3 virulence | Enlarged spleens and LNs: generally well populated with lymphocytes although often some inflammatory debris and neutrophil influx; large fleshy primary lesions with scabbing at the surface; subcutaneous oedema in ventral neck region or sternum. Intense interstitial inflammation in testes with destruction of tubules; survivor had almost complete testicular atrophy at d26. |
| Yorkshire Col:  Grade 2 virulence | Haemorrhage and bacteria in lymph nodes in the earliest death d12. The others were more non-specific with lymphocyte depletion and neutrophil influx in LNs and spleen and orchitis/epididymitis with intense neutrophil infiltration. One animal that died at d14 had patchy interstitial pneumonia. Primary lesions: hypertrophy, hyperplasia and degeneration of epidermal cells with some limited disruption to underlying dermis. |
| Yorkshire 135:  Grade 1 virulence | Liver swollen in some animals; 5 had some pulmonary oedema but this was not present in the earliest death. One animal had foci of coccoid bacteria in liver and LN with no cellular inflammatory response. Spleen and LNs depopulated. Primary lesions: hypertrophy and hyperplasia of epidermal cells with vacuolation and degeneration. Little pathology in underlying dermis. |
