# Supplementary material for: Genomic and phenotypic characterization of myxoma virus from Great Britain reveals multiple evolutionary pathways distinct from those in Australia
Source: PLoS Pathog. 2017 Mar 2;13(3):e1006252. doi: 10.1371/journal.ppat.1006252 (PMC5349684; doi:10.1371/journal.ppat.1006252)
Supplement: S6 Table — (A). Virus titres in Lausanne infected rabbits at day 12 after infection. (B). Virus titres in tissues at autopsy for UK modern isolates. (DOCX) [file ppat.1006252.s008.docx]

**S6 Table.** A. Virus titres in Lausanne infected rabbits at day 12 after infection.

| **Rabbit ID** | **Primary** | **Lung** | **Spleen** | **Liver** | **Lymph node** |
| --- | --- | --- | --- | --- | --- |
| 55 | ^1^8.89 | 6.00 | 4.18 | 3.89 | <2.4 |
| 56 | 7.25 | 4.30 | <2.18 | <2.18 | 4.32 |
| 57 | 7.71 | 2.34 | 3.43 | 3.25 | 4.20 |
| 58 | 7.20 | 6.11 | 4.84 | 5.11 | 6.91 |
| 59 | 7.25 | 5.69 | 4.40 | 4.49 | 4.46 |
| 60 | 6.80 | ^2^ns | ns | ns | ns |

B. Virus titres in tissues at autopsy for UK modern isolates^a^.

| **Virus** | **Rabbit ID** | **Day PI** | **Primary** | **Lung** | **Spleen** | **Liver** | **Lymph node** |
| --- | --- | --- | --- | --- | --- | --- | --- |
| ^3^1527 (5) | 17 | 20 | 7.6 | nd^4^ | nd | nd | 5.0 |
|  | 28 | 24 | 6.0 | nd | nd | nd | 1.8 |
| 1537 (3/4) | 7 | 11 | 8.9 | 8.4 | 8.6 | 8.4 | 8.6 |
|  | 21 | 13.6 | ns^3^ | 8.6 | ns | 8.6 | ns |
| 1792 (2) | 30 | 12 | 9.1 | 6.8 | 6.1 | 6.4 | 7.5 |
|  | 42 | 13 | 8.1 | 4.5 | 2.7 | 4.3 | 5.8 |
|  | 43 | 16 | 7.1 | 4.7 | 4.0 | 6.1 | 5.0 |
|  | 9 | 19 | 9.1 | 5.0 | 6.1 | 5.3 | 6.3 |
|  | 8 | 20 | 9.1 | 5.8 | 2.8 | 5.5 | 7.2 |
| 2082 (3) | 1 | 15 | 9.5 | 7.7 | 5.1 | 6.1 | 7.5 |
|  | 41 | 18 | 6.2 | 5.1 | 6.2 | 5.3 | 5.2 |
|  | 36 | 19 | 9.3 | 5.5 | 2.3 | 4.6 | 6.2 |
|  | 30 | 20 | 9.0 | 4.4 | 2.0 | 4.6 | 6.2 |
| 2282 (3) | 19 | 13 | 9.1 | 7.6 | 6.6 | 6.8 | 5.8 |
|  | 27 | 13 | ns | 7.5 | 6.1 | 5.7 | 7.8 |
|  | 40 | 18 | 9.4 | 7.4 | 5.2 | 6 | 6.8 |
|  | 2 | 22 | 8.8 | 4.4 | 3.2 | 3.0 | 6.1 |
|  | 3BB | 22 | 9.1 | 5.7 | 5.5 | 4.8 | 6.4 |
|  | 5 | 26 | 9.1 | 5.8 | 5.4 | 4.8 | 7.2 |
| York 127 (2/3) | 23 | 11 | 7.3 | 1.8 | 1.9 | 2.7 | 6.0 |
|  | 35 | 13 | 7.4 | 4.8 | 2.2 | 5.4 | 8.8 |
|  | 4 | 12 | 7.5 | 4.4 | 2.8 | 2.9 | 7.1 |
|  | 26 | 13 | 7.9 | 5.9 | 4.5 | 4.0 | 9.4 |
| York col (2) | 59 | 12 | 8.4 | 7.5 | 6.7 | 6.7 | 6.8 |
|  | 57 | 13 | 8.9 | 7.9 | 6.6 | 9.1 | 7.9 |
|  | 14 | 14 | 8.8 | 7.3 | 5.7 | ns | 6.9 |
|  | 39 | 16 | 8.5 | 5.7 | 5.4 | 4.6 | 6.4 |
|  | 60 | 16 | 7.8 | 6.7 | 7.0 | 7.3 | 8.5 |
|  | 10 | 18 | 8.7 | 6.7 | 6.0 | 5.6 | 6.9 |
| York 135 (1) | 51 | 8.7 | ns | ns | ns | 8.5 | ns |
|  | 56 | 9.7 | ns | 7.5 | ns | 8.2 | ns |
|  | 6 | 11.8 | ns | 7.6 | ns | ns | ns |

^a^ The varying number of rabbits for each virus group is because virus titres were only determined for those rabbits euthanized, were seen to die or were obviously recently dead. Rabbits that died overnight were autopsied but not further sampled. Rabbits that were recovering from infection at the conclusion of the trials were not sampled.

1527 and Yorkshire 127 had the cutaneous nodular disease phenotype; 1537 had an intermediate phenotype; all other isolates had the amyxomatous phenotype

1: pfu/g of tissue; 2: not sampled; 3. Virulence grade; 4: not detected
