# Supplementary material for: Genomic and phenotypic characterization of myxoma virus from Great Britain reveals multiple evolutionary pathways distinct from those in Australia
Source: PLoS Pathog. 2017 Mar 2;13(3):e1006252. doi: 10.1371/journal.ppat.1006252 (PMC5349684; doi:10.1371/journal.ppat.1006252)
Supplement: S7 Table — (DOCX) [file ppat.1006252.s009.docx]

**S7 Table**. Amino acid differences between the Yorkshire lineage viruses.

| Gene | Amino acid position | ^1^York 127  Grade 2/3A | York 135  Grade  1 | York Col  Grade  2 | Function |
| --- | --- | --- | --- | --- | --- |
| *M006L/R* | 201 | G | E | E | Predicted E3 ub ligase |
| *M018L* |  | early stop |  |  | unknown function (VACV *F8L*) |
| *M021L* | 254 | R | R | Q | EV maturation factor (VACV *F12L*) |
| *M021L* | 416 | R | Q | Q | EV maturation factor (VACV *F12L*) |
| *M036L* | 100 | A | V | V | VACV *O1L*; ERK1/2 signal potentiation |
| *M036L* | 121 | E | K | E | VACV *O1L*; ERK1/2 signal potentiation |
| *M040L* | 127 | V | V | I | DNA binding |
| *M114R* | 1086 | T | A | T | RNA pol 132k subunit |
| *M122R* | 127 | S | S | G | EV glycoprotein; doesn't alter glycosylation |
| *M124R* | 131 | Y | Y | C | unknown function |
| *M134R* | 153 | S | P | P | unknown function/structural? |
| *M134R* | 1827 | V | L | L | unknown function/structural? |

1. All 3 Yorkshire lineages have disruptions to the *M009L* ORF (Table 2) but the length of the truncated proteins differ between York 127 and the other 2 isolates.

Yorkshire 127 has the nodular form of disease; Yorkshire 135 and Yorkshire col have the amyxomatous phenotype.
