# Supplementary material for: Genomic and phenotypic characterization of myxoma virus from Great Britain reveals multiple evolutionary pathways distinct from those in Australia
Source: PLoS Pathog. 2017 Mar 2;13(3):e1006252. doi: 10.1371/journal.ppat.1006252 (PMC5349684; doi:10.1371/journal.ppat.1006252)
Supplement: S8 Table — (DOCX) [file ppat.1006252.s010.docx]

**S8 Table.** Amino acid differences between the Perthshire lineage 1 viruses.

| Gene | Amino acid position | ^1^ 1527 Grade 5 | 1537  Grade 3/4 | 1792  Grade 2 | Function |
| --- | --- | --- | --- | --- | --- |
| *^2^M002L/R* | - | early stop | early stop | intact | TNF binding & antiapoptosis; virulence |
| *M009L* | 92 | V | A | A | Predicted E3 Ub ligase; gene is disrupted in all 3 viruses |
| *M034L* | 215 | K | E | E | DNA polymerase |
| *M034L* | 468 | K | K | N | DNA polymerase |
| *M058R* | 195 | T | A | A | Major core protein(VACV *L4R*) |
| *M094R* | 17 | E | E | K | RNA polymerase subunit (rpo 19) |
| *M099L* | 760 | A | A | V | Core protein precursor (VACV *A10L*) |
| *M121R* | 152 | E | E | G | EV glycoprotein (VACV *A33R*) |
| *M135R* | - | early stop | intact | intact | unknown; virulence |
| *M140R* | 406 | E | E | K | Predicted E3 Ub ligase |
| *M148R* | 270 | A | A | T | Predicted Ub ligase; virulence |

1. 1527 has the cutaneous nodular phenotype; 1537 has an intermediate phenotype; 1792 has the amyxomatous phenotype.

2. Shaded genes have been demonstrated to have virulence function
