# Supplementary material for: Genomic and phenotypic characterization of myxoma virus from Great Britain reveals multiple evolutionary pathways distinct from those in Australia
Source: PLoS Pathog. 2017 Mar 2;13(3):e1006252. doi: 10.1371/journal.ppat.1006252 (PMC5349684; doi:10.1371/journal.ppat.1006252)
Supplement: S9 Table — (DOCX) [file ppat.1006252.s011.docx]

**S9 Table.** Amino acid differences between the Perthshire lineage 2 viruses.

| Gene | Amino acid position | ^1^2082  Grade 3 | 2282  Grade 3 | Function |
| --- | --- | --- | --- | --- |
| *M008L/R* | - | early stop | intact | predicted E3 Ub ligase |
| *M020L* | 21 | K | E | Ser/Thr protein kinase |
| *M046L* | 47 | G | D | membrane fusion complex |
| *M046L* | 46 | H | R | membrane fusion complex |

1. Both 2082 and 2282 have the amyxomatous phenotype
