# Supplementary material for: Genomic and phenotypic characterization of myxoma virus from Great Britain reveals multiple evolutionary pathways distinct from those in Australia
Source: PLoS Pathog. 2017 Mar 2;13(3):e1006252. doi: 10.1371/journal.ppat.1006252 (PMC5349684; doi:10.1371/journal.ppat.1006252)
Supplement: S10 Table — (DOCX) [file ppat.1006252.s012.docx]

**S10 Table**. All the MYXV sequences used in the evolutionary analysis.

| **Virus** | **Formal name** | **Geographic origin** | **GenBank Accession** | **Reference** |
| --- | --- | --- | --- | --- |
| SLS/1950 (Moses strain/strain B) | None given | Brazil | JX565574 | [1] |
| Glenfield/1951 | Aust/Dubbo/2-51/1 | Australia | JX565567 | [1] |
| KM13/1952 | Aust/Corowa/12-52/2 | Australia | JX565569 | [1] |
| Uriarra/1953 | Aust/Uriarra/2-53/1 | Australia | JX565577 | [1] |
| SWH/9/92 | Aust/Southwell Hill/9-92/1 | Australia | JX565576 | [1] |
| BRK/1993 | Aust/Brooklands/4-93 | Australia | JX565562 | [1] |
| Bendigo/1992 | Aust/Bendigo/7-92 | Australia | JX565565 | [1] |
| Meby/1991 | Aust/Meby/8-91 | Australia | JX565571 | [1] |
| Gung/1991 | Aust/Gungahlin/1-91 | Australia | JX565568 | [1] |
| Wellington/1991 | Aust/Wellington/1-91 | Australia | JX565582 | [1] |
| BRK/12-2-93/1993 | Aust/Brooklands/2-93 | Australia | JX565563 | [1] |
| BD23/1999 | Aust/Bulloo Downs/11-99 | Australia | JX565584 | [1] |
| BD44/1999 | Aust/Bulloo Downs/12-99 | Australia | KC660079 | [1] |
| BRK/897/1995 | Aust/Brooklands/1-95 | Australia | JX565564 | [1] |
| OB1/406/1994 | Aust/OB1/Hall/3-94 | Australia | JX565573 | [1] |
| OB2/W60/1995 | Aust/OB2/Hall/11-95 | Australia | KC660081 | [1] |
| OB3/Y317/1994 | Aust/OB3/Hall/2-94 | Australia | KC660083 | [1] |
| OB3/1120/1996 | Aust/OB3/Hall/2-96 | Australia | KC660082 | [1] |
| WS1/234/1994 | Australia/Woodstock 1 /3-94 | Australia | JX565578 | [1] |
| WS6/1071/1995 | Aust/Woodstock 6 /11-95 | Australia | JX565580 | [1] |
| WS1/328/1994 | Aust/Woodstock 1 /3-94 | Australia | JX565579 | [1] |
| WS6/346/1995 | Aust/Woodstock 6 /3-95 | Australia | JX565581 | [1] |
| SWH/8-2-93/1993 | Aust/Southwell Hill/2-93 | Australia | JX565575 | [1] |
| SWH/805/1993 | Aust/Southwell Hill/11-93 | Australia | KC660085 | [1] |
| SWH/1209/1996 | Aust/Southwell Hill/2-96 | Australia | JX565583 | [1] |
| Lausanne/1949 | Brazil/Campinas/1949/1 | Brazil | KY548791  (re-sequenced here) | [2, 3] |
| Cornwall/1954 | England/Cornwall/4-54/1 | Cornwall UK | JX565566 | [1] |
| Sussex/1954 | England/Sussex/9-54/1 | Sussex UK | KC660084 | [1] |
| Nottingham attenuated/1955 | England/Nottingham/4-55/1 | Nottingham UK | JX565572 | [1] |
| Spain/6918/1995 |  | Spain | EU552530 | [3] |
| Germany/2604/2004 |  | Germany | KP723389 | [4] |
| Germany/3207/2007 |  | Germany | KP723388 | [4] |
| Germany/FLI-H/2004 |  | Germany | KP723390 | [4] |
| Germany/Munich/1985 |  | Germany | KP723387 | [4] |
| Poland/ZA/1985 |  | Poland | KP723386 | [4] |
| Belfast/1955 | Ireland/Belfast/ 1955 | Ireland | KY548792 | This paper |
| Perthshire/ 1526/2008 | Scotland/Perthshire/ 1526/2008 | Scotland | KY548795 | This paper |
| Perthshire /1527/2008 | Scotland/Perthshire /1527/2008 | Scotland | KY548793 | This paper |
| Perthshire/1529/2008 | Scotland/ Perthshire/1529/2008 | Scotland | KY548796 | This paper |
| Perthshire/1537/2008 | Scotland/ Perthshire/1537/2008 | Scotland | KY548794 | This paper |
| Perthshire/1754/2009 | Scotland/ Perthshire/1754/2009 | Scotland | KY548797 | This paper |
| Perthshire/1756/2009 | Scotland/ Perthshire/1756/2009 | Scotland | KY548798 | This paper |
| Perthshire/1792/2009 | Scotland/ Perthshire/1792/2009 | Scotland | KY548799 | This paper |
| Perthshire/1812/2009 | Scotland/ Perthshire/1812/2009 | Scotland | KY548800 | This paper |
| Perthshire/1818/2009 | Scotland/ Perthshire/1818/2009 | Scotland | KY548801 | This paper |
| Perthshire/2080/2010 | Scotland/ Perthshire/2080/2010 | Scotland | KY548802 | This paper |
| Perthshire/2082/2010 | Scotland/ Perthshire/2082/2010 | Scotland | KY548803 | This paper |
| Perthshire/2256/2011 | Scotland/ Perthshire/2256/2011 | Scotland | KY548806 | This paper |
| Perthshire/2272/2011 | Scotland/ Perthshire/2272/2011 | Scotland | KY548804 | This paper |
| Perthshire/2282/2011 | Scotland/ Perthshire/2282/2011 | Scotland | KY548805 | This paper |
| Perthshire/2409/2012 | Scotland/ Perthshire/2409/2012 | Scotland | KY548807 | This paper |
| Perthshire/2427/2012 | Scotland/ Perthshire/2427/2012 | Scotland | KY548808 | This paper |
| Perthshire/2428/2012 | Scotland/ Perthshire/2428/2012 | Scotland | KY548809 | This paper |
| Perthshire/2524/2013 | Scotland/ Perthshire/2524/2013 | Scotland | KY548810 | This paper |
| Yorkshire/127/2008 | England/Yorkshire/127/2008 | England | KY548811 | This paper |
| Yorkshire/Col/2011 | England/Yorkshire/Col/2011 | England | KY548813 | This paper |
| Yorkshire/135/2009 | England/Yorkshire/135/2009 | England | KY548812 | This paper |

1. Kerr PJ, Rogers MB, Fitch A, DePasse JV, Cattadori IM, Twaddle AC, *et al*. (2013). Genome scale evolution of myxoma virus reveals host pathogen adaptation and rapid geographic spread. *J.Virol*. **87**, 12900-12915.

2. Cameron C, Hota-Mitchell S, Chen L, Barrett J, Cao J-X, Macaulay C, *et al*. (1999). The complete DNA sequence of myxoma virus. *Virology* **264**, 298-318.

3. Morales M, Ramirez MA, Cano MJ, Parraga M, Castilla J, Perez-Ordoyo LI, *et al*. (2009). Genome comparison of a nonpathogenic myxoma virus field strain with its ancestor, the virulent Lausanne strain. *J.Virol*. **83**, 2397-2403.

4. Braun C, Thürmer A, Daniel R, Schultz A-K, Bulla I, Schirrmeier H, *et al*. (2017). Genetic variability of Myxoma virus genomes. *J.Virol*. **91**, e01570-16.
